# Associations between dietary intake and asthma outcomes: Evidence from pooled analysis in two independent multiethnic Asian cohorts

**DOI:** 10.1016/j.jacig.2026.100648

**Published:** 2026-01-20

**Authors:** Jun Jie Lim, Zongxun Huang, Yu Qi Lee, Mary Foong-Fong Chong, Xueling Sim, Mei Hui Liu, Fook Tim Chew

**Affiliations:** a Department of Biological Sciences, Faculty of Science, National University of Singapore, Singapore; b Saw Swee Hock School of Public Health, National University of Singapore and National University Health System, Singapore; c Singapore Institute for Clinical Sciences, Brenner Centre for Molecular Medicine, Agency for Science, Technology, and Research (A∗STAR), Singapore; d Department of Food Science & Technology, Faculty of Science, National University of Singapore, Singapore

**Keywords:** Asthma, dietary intake, epidemiology, food frequency questionnaire, food groups

## Abstract

**Background:**

Asthma is a chronic respiratory condition that poses significant public health challenges worldwide, including Singapore.

**Objective:**

Associations between dietary intake and asthma outcomes were assessed in two independent cohorts: the Singapore/Malaysia Cross-Sectional Genetics Epidemiology Study (SMCGES; n = 12,172) and the Singapore Multi-Ethnic Cohort Phase 2 follow-up (MEC2_T2; n = 12,353).

**Methods:**

Dietary intake was assessed using a 16-food-group food frequency questionnaire (FFQ) in SMCGES, and a validated 163-item population-specific FFQ in MEC2_T2. Asthma status was determined through self-reported physician diagnosis, with recent asthma attacks and inhaler requirements analyzed as clinically relevant phenotypes. Multivariable logistic regression models adjusted for demographic and lifestyle factors were applied with Bonferroni correction, and meta-analyses across 16 food groups were conducted to derive pooled effect estimates and assess between-cohort heterogeneity.

**Results:**

Asthma prevalence was 19.7% in SMCGES and 9.83% in MEC2_T2. Among patients with asthma, 18.8% (SMCGES) and 18.7% (MEC2_T2) experienced recent asthma attacks, while inhaler requirement was lower in SMCGES (5.9%) than MEC2_T2 (18.4%). Fruit (pooled odds ratio [pOR] = 0.65; 95% confidence interval [CI], 0.57-0.74; *P* < .001) and nuts (pOR = 0.90; 95% CI, 0.85-0.95; *P* < .001) intake lowered the odds of asthma, while seafood intake increased the associated odds (pOR = 1.13; 95% CI, 1.07-1.20; *P* < .001). Fruit intake showed moderate heterogeneity (*I*^2^ = 63%; *P* = .10), with no heterogeneity for nuts (*I*^2^ = 0; *P* = .69) or seafood (*I*^2^ = 0, *P* = .56).

**Conclusion:**

Pooled findings across two independent cohorts highlight specific food groups that may influence asthma outcomes.

Asthma is a chronic respiratory condition characterized by inflammation and airway hyperresponsiveness, which impairs normal airflow and makes breathing difficult, affecting millions worldwide.^1^ In Singapore, asthma prevalence is notable, with significant implications due to the associated morbidity, health care utilization, and impact on quality of life.^2^^,^^3^ Although asthma has no cure, management through personalized action plans— incorporating trigger avoidance, preventive medication, and monitoring—can help control symptoms.^4^

Effective asthma management remains a global priority, and diet has emerged as a modifiable factor that may influence asthma risk and clinical expression.^5^, ^6^, ^7^ Nutritional approaches are particularly relevant given their potential to modulate inflammation and immune function, both of which are crucial to asthma pathophysiology. The growing prevalence of asthma, particularly in highly urban and developed regions, has paralleled dietary shifts toward patterns typically low in fruits and vegetables but high in saturated fats and processed foods.^8^ Evidence suggests that diets rich in plant-based foods (fruits, vegetables, grains, and legumes), such as Mediterranean and vegan diets, may reduce asthma risk and improve symptom control through anti-inflammatory and immune-modulating effects.^5^^,^^9^ In contrast, higher intake of saturated fats and dairy have been associated with worsened asthma outcomes.^10^ Although nutrients like antioxidants, fiber, polyunsaturated fatty acids, and vitamin D have shown some positive influences on immune responses central to asthma’s pathophysiology, further interventional research is needed to substantiate dietary recommendations for asthma prevention and management.^10^, ^11^, ^12^, ^13^

Asthma is a heterogenous condition with varying clinical presentations and disease activity. Although emerging studies have explored diet–asthma associations, most have focused on Western pediatric populations, with limited evidence from Asian adults despite distinct dietary intake, lifestyle, and asthma prevalence in this region.^14^^,^^15^ The International Study of Asthma and Allergies in Childhood (ISAAC) established the utility of a validated, simplified, semiquantitative food frequency questionnaire (FFQ) for identifying food–asthma associations in children and adolescents.^16^ Building on this framework, our earlier analysis of the Singapore/Malaysia Cross-Sectional Genetics Epidemiology Study (SMCGES) allergic cohort identified similar diet–asthma associations among young Chinese adults, where frequent consumption (≥3 times per week) of pasta, butter, and margarine was associated with higher odds of asthma and related allergic diseases such as atopic dermatitis, while greater intake of pulses and probiotic drinks was associated with lower asthma odds.^2^^,^^17^, ^18^, ^19^, ^20^

Extending beyond these earlier findings, the present study aimed to strengthen the evidence for diet–asthma associations using another independent large Singaporean cohort: the Multi-Ethnic Cohort Phase 2 follow-up (MEC2_T2), which used a comprehensive 163-item semiquantitative FFQ specifically validated for Singapore’s multiethnic population. By pooling data from SMCGES and MEC2_T2 cohorts, we evaluated the robustness and reproducibility of diet–asthma associations across independent adult populations with differing dietary assessment tools. Recognizing the heterogeneity of asthma, our study focused on physician-diagnosed asthma and further differentiated current patients with asthma by recent asthma attacks and inhaler requirements—two important indicators of disease activity and management needs. This cross-cohort comparison provides a unique opportunity to assess the consistency of associations across distinct yet comparable Asian adult populations in Singapore, enhancing the interpretability and clinical relevance of dietary influences on asthma beyond disease prevalence alone.

## Methods

### Study cohorts

SMCGES recruited participants aged ≥18 years from campuses of the National University of Singapore (Singapore), Sunway University (Malaysia), and Universiti Tunku Abdul Rahman (Malaysia). Recruitment was conducted annually from 2005 to 2023. Informed consent was obtained from the participants. For participants aged <21 years, parental consent was mandatory and was obtained for participation. The study was conducted according to the Declaration of Helsinki and Good Clinical Practices. Detailed information on the SMCGES cohort has been previously published.^17^, ^18^, ^19^, ^20^, ^21^, ^22^, ^23^, ^24^, ^25^, ^26^ SMCGES used a standardized, investigator-administered questionnaire based on the ISAAC protocol to collect data on personal medical histories, socioeconomic factors, lifestyle, dietary intake, and anthropometrics.^27^ Basic demographic details, including age (years), sex, ethnicity, income category (Singapore dollars), and body mass index (BMI) (Asian classification), were collected, along with lifestyle information on alcohol consumption (never, occasional, or frequent) and smoking status (nonsmoker, ex-smoker, or current smoker). Because asthma prevalence patterns in Malaysia differ from those in Singapore as a result of potential regional factors that remain poorly understood, only data collected in Singapore from SMCGES were included here to allow for direct comparison with another Singapore-based cohort. From the initial cohort of 14,002 participants surveyed in Singapore, we excluded participants with missing data on age and sex (n = 158), anthropometric measurements (n = 1,273), and income information (n = 399). A final sample of 12,172 participants (mean ± SD age, 22.4 ± 5.8 years) were included in the current analysis (see Fig E1 in this article’s Online Repository available at www.jaci-global.org).

The MEC2_T2 study, conducted from 2013 to 2016, included Singaporean citizens and permanent residents aged 21 to 75 from the 3 major Asian ethnic groups: Chinese, Indian, and Malay.^28^ The participants were recruited at baseline between 2011 and 2015 and were invited for a follow-up visit between 2016 to 2020. Sociodemographic factors, personal medical history, lifestyle factors, and body composition assessments have been previously detailed in Lee et al.^29^ From the initial cohort of 19,269 participants surveyed, we excluded participants with missing data on anthropometric measurements (n = 5,232), income information (n *=* 1,655), and dietary intake (n = 4); we also excluded those who did not report an asthma diagnosis (n = 25). A final sample of 12,353 participants from MEC2_T2 (mean ± SD age, 49.1 ± 13.3 years) was included in the current analysis (Fig E1).

### Ethics approval and consent

This study was conducted in accordance with the principles of the Declaration of Helsinki and Good Clinical Practices, and in compliance with local regulatory requirements. The Singapore cohort of SMCGES was conducted on the National University of Singapore campus annually between 2005 and 2022, under the approval of the institutional review board (approvals NUS-07-023, NUS-09-256, NUS-10-445, NUS-13-075, NUS-14-150, and NUS-18-036) and by the Helsinki Declaration. Before data collection, all participants or their guardians signed an informed consent form. The MEC2_T2 study was conducted under the approval of the institutional review board (approval NUS-LB-16-125). Our study was approved by the institutional review board (approval NUS-IRB-2023-700).

### Defining asthma outcomes

In the SMCGES cohort, asthma data were collected using validated ISAAC guidelines suitable for both children and adults, defining “ever asthma” as doctor-diagnosed asthma verified by the specific question, “Have you ever had asthma?” A doctor’s diagnosis, guided by Global Initiative for Asthma standards, ensures accurate assessment through medical history, physical examination, and standardized criteria, reducing misclassification and ensuring consistency across cases.^30^^,^^31^ Among the 12,172 participants, 2,394 (19.7%) were identified to have asthma. Participants who did not affirm doctor-diagnosed asthma were classified as nonasthma controls (n = 9,778, 80.3%), serving as the reference group in the subsequent logistic regression analysis. Of the 2,394 asthma cases, recent attacks were assessed by the question, “In the past 12 months, how often, on average, have you experienced asthma attacks during the day or night?” Those who responded “not at all” (n = 1,944 , 81.2%) were classified as experiencing no attacks, while all other responses (“less frequently than monthly,” “1-3 times a month,” “1-3 times a week,” “4-6 times a week,” or “every day”) were classified as experiencing attacks (n = 450, 18.8%). Similarly, recent inhaler requirement was assessed among patients with asthma using the question, “In the past 12 months, how many times on average have you used your inhaler?” Participants who responded “never” were categorized as having no inhaler requirement (n = 2,335, 97.5%), while all other affirmative responses were categorized as requiring inhalers (n = 59, 2.5%) (Fig E1). These phenotypes were included as clinically relevant indicators of recent asthma activity and management practices, consistent with established population-based respiratory research.

A self-reported question, “Have you ever been told by a Western-trained doctor that you have asthma,” was used to identify doctor-diagnosed asthma for “ever asthma” presentation. Among the 12,353 participants in the MEC2_T2 population, 1,214 (9.83%) were identified with asthma. Among 1,214 asthma cases, recent attacks were assessed using the question, “During the last 12 months, have you had an episode of asthma or an asthma attack?” Of the 1,214 participants with asthma, 227 (18.7%) reported having an attack and 987 (81.3%) reported no attacks. Recent inhaler requirement was assessed with the question, “Over the past 1 month, on average, how many times per week do you use your inhaler medication for quick relief of asthma symptoms?” A total of 223 (18.4%) reported inhaler requirement, while 991 (81.6%) reported no such requirement (Fig E1).

### Dietary intake assessments

In the SMCGES cohort, dietary intake across 16 food groups was assessed using a validated semiquantitative FFQ adapted from the ISAAC phase 3 study.^32^ Participants reported their habitual intake over the past 12 months, answering, “In the past 12 months, how often, on average, did you eat or drink the following: Meat (eg, beef, lamb, chicken, pork); Seafood (including fish); Fruits; Vegetables (green and root); Pulses (peas, beans, lentils); Cereals (including bread); Rice; Butter; Margarine; Nuts; Potatoes; Milk; Eggs; Burgers/fast food (collectively known as fast food); Yakult/Vitagen/similar yogurt drinks (collectively known as probiotic drinks)?” Intake frequencies were recorded as never/occasionally, once or twice weekly, or most/all days, corresponding to servings per week. These food groups represent primary sources of energy and nutrients globally, facilitating robust international comparisons and accurate dietary assessments related to nutritional intake and allergic disease.^16^^,^^31^^,^^32^ Standardized average portion sizes were used to guide participants, minimizing interpretation errors, while direct administration by trained investigators reduced recall bias and misunderstandings.

In MEC2_T2, dietary intake was assessed using a semiquantitative 163-item FFQ validated for Singapore’s multiethnic population. This FFQ has demonstrated high reproducibility and validity through significant correlations with 24-hour dietary recalls and relevant biomarkers, serving as a robust tool for evaluating dietary exposures in Singapore’s diverse population.^33^ Intake frequency for each food item was recorded per day and converted to servings per week for harmonization with the FFQ used in SMCGES. Items were then grouped into the same 16 food groups, and total group intake was calculated by summing weekly servings across items within each group (see Table E1 in the Online Repository available at www.jaci-global.org).

For both cohorts, food group exposures were categorized into 3 frequency-based levels: <1 serving per week (never or only occasionally), 1-2 servings per week (once or twice per week), and ≥3 servings per week (most or all days). These frequencies were based on the established ISAAC framework, reflecting habitual rather than single-point dietary intake.^32^ Detailed intake distributions by asthma phenotype are provided in Tables E2-E4 in the Online Repository available at www.jaci-global.org.

### Assessment of covariates

Covariates included in the analysis were age (years), sex, ethnicity, income category (Singapore dollars), alcohol intake, smoking status, and BMI (Asian classification). These variables were adjusted in the multivariable models to account for potential confounding effects on the diet–asthma associations guided by their established relevance in previous epidemiologic studies and their potential influences on the outcome of interest.^2^^,^^34^

### Statistical analysis

Logistic regression was used to model the association between presence of asthma and dietary intake. A likelihood ratio test was further conducted to obtain the nested *P* value for the overall effect of dietary intake on asthma risk. Bonferroni correction was used to account for multiple testing, with the adjusted *P* value cutoff for significance set at .003125 = .05/16. We utilized the DerSimonian-Laird random effects method to account for between-study heterogeneity^35^ while combining the effect size of the diet–asthma association for the 16 food groups in both SMCGES and MEC2_T2. Pooled odds ratios (pOR) were obtained from the meta-analysis. Heterogeneity was assessed using the heterogeneity *P* < .05 and *I*^2^ index, with values exceeding 50% indicating significant heterogeneity among the studies. All statistical analyses, including meta-analysis, were conducted by R v2024.09.0-375 software (www.r-project.org, 2024).^36^

## Results

### Demographic comparisons between SMCGES and MEC2_T2 populations

Table I lists key demographic and lifestyle factors of the MEC2_T2 (n = 12,353) and SMCGES (n = 12,172) cohorts. Asthmatic patients were younger than nonasthmatic controls in MEC2_T2 (case, 44.1 ± 13.8; noncase, 49.6 ± 13.2 years) and SMCGES (case, 20.7 ± 6.0; noncase, 22.8 ± 5.7 years). There were significant ethnic differences, with a higher proportion of Malays and Indians among patients with asthma in both cohorts. In both cohorts, patients with asthma were more likely to be overweight. In MEC2_T2, patients with asthma were more likely to be ever or current smokers (28.2%), while no significant differences in smoking status were observed in SMCGES. Drinking status was associated with asthma in both cohorts.

**Table I:** Demographic characteristics of study cohorts

Characteristic	MEC2_T2 (n = 12,353)			SMCGES (n = 12,172)		
	Nonasthmatic controls	Asthma cases	*P* value	Nonasthmatic controls	Asthma cases	*P* value
No. of subjects	11,139	1214		12,172	2394	
Age, mean ± SD	49.6 ± 13.2	44.1 ± 13.8	<2.20 × 10^−1^^6^	22.8 ± 5.7	20.7 ± 6.0	<2.20 × 10^−1^^6^
Sex			1.43 × 10^−1^			<2.20 × 10^−1^^6^
Male	5012 (45.0)	573 (47.2)		4039 (41.3)	1216 (50.8)	
Female	6127 (55.0)	641 (52.8)		5739 (58.7)	1178 (49.2)	
Ethnicity			3.48 × 10^−1^^8^			2.62 × 10^−^^34^
Chinese	7804 (70.1)	724 (59.6)		8616 (88.1)	1997 (83.4)	
Malay	1354 (12.2)	240 (19.8)		189 (1.9)	157 (6.6)	
Indian	1981 (17.8)	250 (20.6)		537 (5.5)	159 (6.6)	
Other	0	0		436 (4.5)	81 (3.4)	
BMI (Asian classification)			2.09 × 10^−^^5^			2.88 × 10^−^^2^
Underweight (<18.0 kg/m^2^)	544 (4.9)	41 (3.4)		1821 (18.6)	481 (20.1)	
Healthy (18.0-23.0 kg/m^2^)	3837 (34.5)	356 (29.3)		6157 (63.0)	1437 (60.0)	
Overweight (>23.0 kg/m^2^)	6758 (60.7)	817 (67.3)		1800 (18.5)	476 (19.9)	
Average monthly total household income (Singapore dollars)			7.77 × 10^−1^			4.12 × 10^−^^4^
Less than S$2000	2281 (20.5)	234 (19.3)		2198 (22.5)	463 (19.3)	
S$2000 to S$3999	2516 (22.6)	284 (23.4)		3180 (32.5)	736 (30.7)	
S$4000 to S$5999	2376 (21.3)	250 (20.6)		1875 (19.2)	498 (20.8)	
More than S$6000	3966 (35.6)	446 (36.7)		2525 (25.8)	697 (29.1)	
Smoking status			5.99 × 10^−^^9^			4.13 × 10^−1^
Never smoker	8841 (79.4)	871 (71.7)		9498 (97.1)	2325 (97.1)	
Ever smoker	939 (8.4)	139 (11.4)		158 (1.6)	45 (1.9)	
Current smoker	1359 (12.2)	204 (16.8)		122 (1.2)	24 (1.0)	
Alcohol consumption			2.60 × 10^−^^3^			5.42 × 10^−^^9^
Nondrinker	8295 (74.5)	869 (71.6)		4795 (49.0)	1336 (55.8)	
Occasional	2789 (25.0)	331 (27.3)		4767 (48.8)	999 (41.7)	
Drinker	55 (0.5)	14 (1.2)		216 (2.2)	59 (2.5)	

Data are presented as nos. (%) unless otherwise indicated. Asthma was defined as doctor-diagnosed asthma according to Global Initiative for Asthma guidelines. Chi-square *P* values were considered statistically significant at *P* < .05. Student *t* test was used to analyze difference in age, while chi-square test was used to examine difference in other categorical variables (sex, ethnicities, BMI, smoking status, and alcohol consumption).

### Association between 16 food groups and asthma outcomes

**Fig 1:**
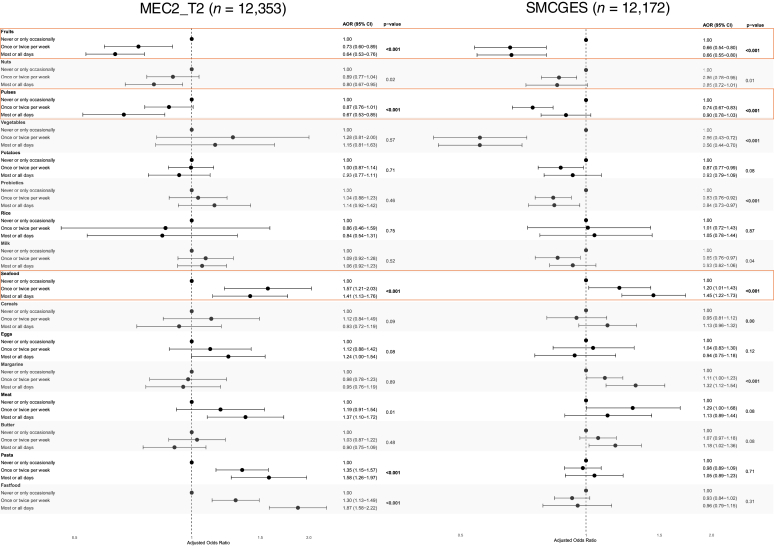
aOR illustrating association between 16 food types and asthma presentation in **(A)** MEC2_T2 (n = 12,353) and **(B)** SMCGES (n = 12,172) cohorts. *Dotted line* at aOR = 1.00 serves as reference. Results are presented as aORs (95% CIs) and *P* values. Multivariable analysis was adjusted for age (years), sex, BMI (Asian classification), alcohol intake, smoking status, income category, and ethnicity. Bonferroni correction was used to account for multiple testing, with adjusted *P* value cutoff for significance set at .003125 = .05/16.

Fig 1 shows the adjusted odds ratio (aOR) comparing the association between dietary intake across 16 food groups and asthma presentation in the MEC2_T2 and SMCGES cohorts. Figs E2 and E3 in the Online Repository available at www.jaci-global.org depict the corresponding aOR for recent asthma attacks and inhaler requirement, respectively. Increased fruit consumption was consistently associated with reduced odds of asthma across both cohorts (*P* < .001). In MEC2_T2, frequent (most or all days) fruit intake significantly lowered the odds of asthma (aOR = 0.64; 95% confidence interval [CI], 0.53-0.76). A similar inverse association was observed in SMCGES for fruit intake (aOR = 0.66; 95% CI, 0.55-0.80). The intake of pulses also showed an inverse association with asthma (*P* < .001). In MEC2_T2, frequent pulses intake showed a protective association (aOR = 0.67; 95% CI, 0.53-0.85), while only occasional (once or twice per week) intake of pulses was associated with lower odds of asthma in SMCGES (aOR = 0.74; 95% CI, 0.67-0.83). Intake of pulses was also associated with protective odds for recent asthma attacks (*P* < .001) only in SMCGES (Fig E2). In contrast, frequent seafood consumption increased the odds of asthma in both cohorts (*P* < .001), with MEC2_T2 (aOR = 1.41; 95% CI, 1.13-1.76) and SMCGES (aOR = 1.45; 95% CI, 1.22-1.73) showing positive associations. Although frequent nuts intake demonstrated a nominal protective association with asthma in both cohorts, this association did not remain statistically significant after correction for multiple comparisons. Interestingly, nut intake was associated with protective odds for recent asthma attacks (*P* < .001) in the SMCGES cohort (Fig E2).

Several food groups were associated with asthma in only one of the two cohorts. Fast food intake was associated with increased odds of asthma in MEC2_T2 (*P* < .001), where occasional intake was sufficient to increase odds of asthma (aOR = 1.30; 95% CI, 1.13-1.49), and the odds increased further with frequent consumption (aOR = 1.87; 95% CI, 1.58-2.22). However, this association was not observed in SMCGES (*P* = .31) for occasional (aOR = 0.93; 95% CI, 0.84-1.02) or frequent (aOR = 0.96; 95% CI, 0.79-1.15) fast food intake. Similarly, pasta intake showed a significant association with asthma in MEC2_T2 (*P* < .001) but not SMCGES (*P* = .71). While meat consumption was initially associated with increased odds of asthma in MEC2_T2 (*P* = .01), this association was no longer significant after Bonferroni correction. In SMCGES, meat consumption was not significantly associated with asthma (*P* = .08).

Interestingly, the association between vegetable intake and asthma varied between the two cohorts. In MEC2_T2, vegetable consumption was not associated with asthma (*P* = .57). However, vegetable intake showed a protective association with asthma in SMCGES (*P* < .001). Similarly, frequent probiotic consumption lowered the odds of asthma in SMCGES (*P* < .001) but not in MEC2_T2 (*P* = .46). Margarine intake increased the odds of asthma significantly in SMCGES (*P* < .001) but not in MEC2_T2 (*P* = .89).

### Pooled analysis

**Fig 2:**
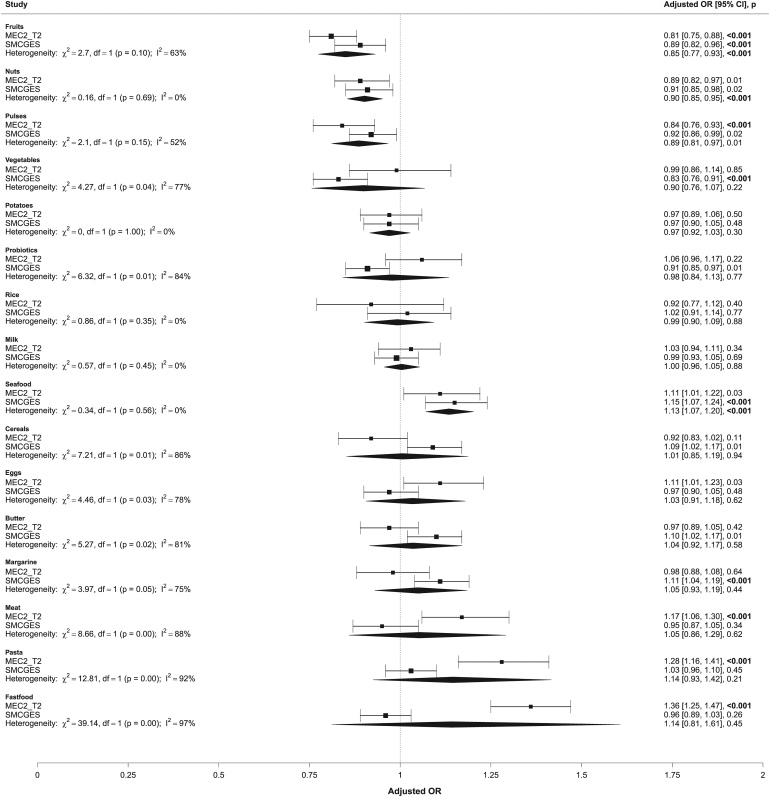
Forest plot depicting association between asthma presentation and dietary intake across 16 food groups pooled from MEC2_T2 (n = 12,353) and SMCGES (n = 12,172) cohorts.

Dietary intake across 16 food groups was assessed in a pooled analysis of both cohorts for asthma presentation (Fig 2) and its clinical phenotypes involving recent asthma attacks (see Fig E4 in the Online Repository available at www.jaci-global.org) and requirement for inhalers (see Fig E5, also in the Online Repository). Fruit intake was significantly associated with lowered odds of asthma (pOR = 0.85; 95% CI, 0.77-0.93; *P* < .001), indicating consistent protective effects across cohorts. Although moderate heterogeneity was observed for fruit intake, it was not statistically significant (*I*^2^ = 63%; *P* = .10). Nuts intake was also significantly associated with lowered odds of asthma after adjusting for multiple comparisons (pOR = 0.90; 95% CI, 0.85-0.95; *P* < .001), with no heterogeneity observed (*I*^2^ = 0.00%; *P* = .69). Each cohort showed nominal significance. Although pulses also showed a protective association, with the pooled estimate indicating lower odds of asthma (pOR = 0.89; 95% CI, 0.81-0.97; *P* = .01), this association did not cross the Bonferroni correction threshold. Finally, seafood intake was significantly associated with increased odds of asthma (pOR = 1.13; 95% CI, 1.07-1.20; *P* < .001), with no evidence of heterogeneity (*I*^2^ = 0.00%; *P* = .56). These associations between fruits, nuts, pulses, and seafood were not significant for neither recent attacks or use of inhalers among patients with asthma.

While associations between fast food, pasta, meat, probiotic, and vegetables intake with asthma were observed in individual cohorts, these associations were not consistent in the pooled analysis. Substantial heterogeneity was observed between cohorts for fast food (*I*^2^ = 97%, *P* < .001), pasta (*I*^2^ = 92%, *P* < .001), probiotics (*I*^2^ = 84%, *P* < .001), and vegetables (*I*^2^ = 77%, *P* = .04). No significant associations were observed between the remaining food groups (ie, potatoes, rice, milk, cereals, eggs, butter, and margarine) and asthma in the pooled analysis.

## Discussion

Our findings showed that associations between fruit, nuts, and seafood intake and asthma were consistent across the MEC2_T2 and SMCGES cohorts despite differences in dietary assessment methods. Several food groups such as fast food, pulses, and vegetables showed some heterogeneity in their associations with asthma. The observed age-related differences in asthma prevalence between cohorts likely reflect differences in age distributions, as asthma typically peaks in childhood and adolescence before declining with age.^3^^,^^34^ Early-life respiratory infections, particularly respiratory syncytial virus and human rhinovirus, have been shown to increase asthma susceptibility through long-term effects on airway inflammation and altered immune maturation.^37^^,^^38^ Although infection history was not captured, both cohorts were community based and were not selected by infection or asthma status; such early-life exposures, if present, are therefore expected to be randomly distributed and unlikely to confound the observed diet–asthma associations. The age-related decline in asthma prevalence is more plausibly explained by physiologic changes in airway and immune function with maturation.^39^ Nevertheless, future studies incorporating infection history would be helpful to clarify potential virus–diet interactions in asthma risk.

Despite a similar proportion of participants having recent asthma attacks in both cohorts (∼18%), inhaler requirement was substantially lower in the younger SMCGES cohort (5.9%) compared to the older MEC2_T2 cohort (18.4%). This discrepancy likely reflects age-related differences in disease management and health care engagement rather than disease severity alone. As detailed in our previous SMCGES analysis on asthma phenotypes, these young adults tended to experience milder or intermittent symptoms with better control, and they were less likely to seek medical care or require adherence to prescribed controller therapies.^17^ In contrast, older adults likely experience longer disease duration and greater comorbidity burden, prompting a higher treatment adherence and inhaler requirement compared to a younger population.^40^ These findings suggest that comparable attack frequencies may mask disparities in disease recognition and management practices, underscoring the importance of strengthening public awareness and education on asthma control and appropriate treatment use across all age groups.

A critical strength of this study is the detailed quantification of dietary exposures using frequency-based data across both cohorts, harmonized into weekly servings to reflect habitual dietary intake rather than single-point dietary intake. This approach minimizes measurement error, enables cross-cohort comparisons despite differing FFQ designs, and supports dose–response evaluation, which is relevant to chronic conditions such as asthma. Supporting data from a subset of SMCGES participants with both FFQ and 3-day food diaries showed substantial agreement (Cohen κ > 0.6) for key food groups, reinforcing the validity of the FFQ in capturing habitual intake. While broad frequency categories may not capture finer intake variations, the large sample sizes (n > 12,000) provide robust estimates, and the observed associations are largely aligned with prior literature,^5^^,^^7^^,^^9^^,^^15^^,^^16^^,^^32^ supporting biological plausibility. Although these findings are limited to only two cohorts, they provide actionable insights, and future studies should systematically include additional cohorts—especially within Asian adult populations—and leverage longitudinal data (eg, GUSTO [Growing Up in Singapore Towards healthy Outcomes]) to further validate and strengthen causal inference in diet–asthma research.^41^

Because the FFQs capture habitual dietary intake over several months, these data reflect long-term exposure rather than short-term dietary variation. These associations observed therefore reliably represent habitual dietary intake related to asthma activity. While the ISAAC FFQ is relatively simple compared to more detailed instruments like the 163-item FFQ used in MEC2_T2, many of the associations between food groups were consistently observed. The ISAAC FFQ remains a practical solution for researchers conducting large-scale, time-limited studies, offering a time-efficient and cost-effective means of dietary assessment across diverse populations. However, further validation across varied cohorts, including those with different disease outcomes and cultural backgrounds, is necessary to strengthen its reliability. Beyond individual food intake, deriving relevant dietary indices such as the Dietary Inflammatory Index provides an additional layer of insight into how overall dietary patterns may contribute to asthma risk.^42^

Previous findings from the SMCGES cohort demonstrated some consistent associations between dietary intake and allergic sensitization.^17^ In this pooled cross-cohort study, seafood intake was positively associated with asthma, whereas higher fruit and nut intake showed inverse associations, suggesting these diet–asthma associations were not solely allergy mediated and remained relevant at the population level. Importantly, these associations were observed in doctor-diagnosed asthma and clinically relevant phenotypes such as recent attacks and inhaler requirement, underscoring their practical relevance for public health and clinical guidance. Ongoing work in a subset of SMCGES participants includes serologic screening and component-resolved diagnostics for food-specific IgE against key allergens such as *Arachis hypogaea* (peanut) and seafood (crustacean, molluscs, fish), which will provide more precise insights into IgE-mediated food sensitization patterns contributing to asthma risk.^43^ While biomarker data were not yet available to define mechanistic endotypes, the well-characterized clinical features in these cohorts provide a strong foundation for future in-depth phenotyping. Planned studies in SMCGES are also evaluating peripheral blood mononuclear cells and transcriptomic profiles to classify more specific asthma phenotypes, including eosinophilic and molecular endotypes, complementing population-level analyses of modifiable lifestyle factors, including diet.

By pooling data from the MEC2_T2 and SMCGES cohorts, this study enhances statistical power and allows a more comprehensive assessment of food-specific risk- and protective-associated factors for asthma that accounts for intercohort variability. This approach contrasts with many previous studies that focus primarily on pediatric populations that may not capture dietary influences in a broader adult demographic.^44^, ^45^, ^46^ Our findings are broadly consistent with the existing literature, particularly the protective association between fruit intake and reduced asthma risk, likely attributed to their antioxidant, anti-inflammatory, and immune-modulating properties.^47^, ^48^, ^49^ Although fruit intake was significantly associated, vegetable intake did not show the same inverse association, potentially because of the higher bioavailability of bioactive compounds in fruits than vegetables.^49^ Fruits such as oranges are rich in vitamin C and anti-inflammatory flavonoids, which may reduce oxidative stress and airway inflammation, thereby alleviating asthma symptoms.^50^^,^^51^ Beyond fruits, the inclusion of more plant-based foods, such as vegetables and whole grains, may also contribute to lower asthma risk by improving diet quality and reducing the intake of proinflammatory foods.^9^^,^^23^^,^^52^, ^53^, ^54^, ^55^ Although nuts are common allergens for some individuals, our study found an inverse association between nut intake and asthma risk. This protective association may be attributed to their rich antioxidant content and potential synergistic effects when incorporated into a Mediterranean-style dietary pattern.^56^^,^^57^ An initial inverse association between intake of pulses and asthma was observed in this study, but it did not remain statistically significant after Bonferroni correction. Nonetheless, given the known anti-inflammatory and antioxidant properties of pulses, their potential role in asthma prevention should not be underestimated and warrants further investigation.^58^

In contrast, our study found a positive association between seafood intake and asthma, but this finding should be interpreted with caution. Evidence from prior epidemiologic studies remains mixed; meta-analytic data suggest a modest protective effect of fish or fish oil intake on asthma risk,^59^and pooled analyses of large European and US birth cohorts reported no increased risk with maternal fish or seafood consumption during pregnancy.^60^ However, specific seafood subtypes, particularly shellfish, contain allergenic proteins such as tropomyosin that can provoke respiratory symptoms in hypersensitized individuals or individuals with occupational exposure.^61^^,^^62^ Because our analysis did not distinguish between finfish and shellfish or account for food-specific sensitization, the observed association likely reflects population heterogeneity rather than a direct causal link. Ongoing work within a subset of SMCGES participants is currently examining seafood-specific IgE responses to clarify the contribution of shellfish and other seafood subtypes to asthma risk.

While fast food intake was significantly associated with asthma in the MEC2_T2 cohort, this association was not observed in the SMCGES cohort, despite an earlier ISAAC study identifying fast food as an associated risk factor for asthma.^32^ Prior research has focused mainly on children and adolescents.^32^^,^^63^ A possible explanation for this discrepancy lies in the age differences between cohorts: SMCGES participants were predominantly young adults (mean age ∼22 years), while MEC2_T2 included primarily older adults (mean age ∼50 years). This may reflect cumulative dietary exposure, age-related metabolic changes, or increased susceptibility to proinflammatory effects.^64^ However, given the cross-sectional design, these hypotheses warrant further investigations using longitudinal data. Fast food intake frequency (ie, consuming fast food on most or all days) was also relatively higher in MEC2_T2 (∼12%) than SMCGES (∼6%), suggesting that intake quantity may also partially explain the differential findings. Overall, these findings highlight the need for future studies to explore how the relationship between fast food and asthma evolves across different life stages. Age-specific factors should be further investigated to clarify the long-term respiratory effects of fast food consumption.^64^

We acknowledge that foods are rarely consumed in isolation and that subtypes within food groups, such as shellfish versus finfish or peanuts versus other nuts, may differ in allergenic potential. In this study, seafood and nuts were assessed as composite categories to ensure cross-cohort comparability and sufficient statistical power, which is a common approach in nutritional epidemiology. While such grouping may mask subtype-specific effects, prior SMCGES correlation analyses showed minimal overlap between most food groups (eg, seafood showed negligible correlation with fruits, nuts, and pulses),^23^ supporting the validity of independent group-level analyses. The observed associations—higher seafood intake and higher asthma odds, and higher fruit and nut intake and lower asthma odds— were consistent across cohorts and aligned with existing literature, reinforcing biologically plausible population-level relationships. However, our study’s cross-sectional design limits causal inferences, as dietary intake and asthma outcomes were measured concurrently. Future studies should adopt detailed dietary records or 24-hour recalls to distinguish specific food subtypes (eg, finfish vs shellfish, peanuts vs tree nuts), integrate allergen-specific IgE or metabolomic biomarkers to validate exposures, and leverage longitudinal cohorts such as GUSTO^41^ to establish temporal and causal relationships between diet and asthma risk.

In conclusion, this study highlights the significant associations between dietary intake and asthma outcomes, emphasizing the inverse association between fruit and nut intake and the potential risks associated with high seafood intake. The pooled analysis across two independent cohorts underscores the reliability of the ISAAC 16-food group FFQ as a practical tool for large-scale dietary assessment, despite some observed heterogeneity in specific food groups. These findings provide valuable insights into diet–asthma relationships, supporting the need for longitudinal research and refined dietary assessments to inform public health strategies for asthma prevention.

## Disclosure Statement

The Multi-Ethnic Cohort Phase 2 follow-up (MEC2_T2) study is supported by infrastructure funding from the Singapore Ministry of Health (Population Health Metrics and Analytics), National University of Singapore, and National University Health System, Singapore. For the Singapore/Malaysia Cross-Sectional Genetics Epidemiology Study (SMCGES), F.T.C. received grants from the National University of Singapore (N-154-000-038-001 [E-154-00-0017-01], C141-000-077-001 [E-141-00-0096-01]), Singapore Ministry of Education Academic Research Fund (R-154-000-191-112, R-154-000-404-112, R-154-000-553-112, R-154-000-565-112, R-154-000-630-112, R-154-000-A08-592, R-154-000-A27-597, R-154-000-A91-592, R-154-000-A95-592, R154-000-B99-114), Biomedical Research Council (BMRC) (Singapore) (BMRC/01/1/21/18/077, BMRC/04/1/21/19/315, BMRC/APG2013/108), Singapore Immunology Network (SIgN-06-006, SIgN-08-020), National Medical Research Council (NMRC) (Singapore) (NMRC/1150/2008, OFIRG20nov-0033, MOH-001636 [OFLCG23may-0038, A-8002641-00-00]), National Research Foundation (NRF) (Singapore) (NRF-MP-2020-0004), Singapore Food Agency (SFA) (SFS_RND_SUFP_001_04, W22W3D0006), Singapore’s Economic Development Board (EDB) (A-8002576-00-00), and the Agency for Science Technology and Research (A∗STAR) (Singapore) (H17/01/a0/008, APG2013/108). This research was supported by the NRF (Singapore) under its Open Fund–Large Collaborative Grant (MOH-001636, A-8002641-00-00) and administered by the Singapore Ministry of Health’s NMRC. The funding agencies had no role in the study design, data collection and analysis, decision to publish, or preparation of the report.

Availability of data and materials: Data concerning the SMCGES cohort will be shared on reasonable request from F. T. Chew. Researchers can request data from the MEC2_T2 study for scientific purposes through an online application process (blog.nus.edu.sg/sphs/data-and-samples-request/). Data will be shared through an institutional data-sharing agreement.

Disclosure of potential conflict of interest: F. T. Chew reports grants from the National University of Singapore, Singapore Ministry of Education Academic Research Fund, Singapore Immunology Network, NMRC, BMRC, NRF, SFA, Singapore’s EDB, and A∗STAR during the conduct of the study; and consulting fees outside the current report from Sime Darby Technology Centre, First Resources, Genting Plantation, Olam International, Musim Mas, and Syngenta Crop Protection. The rest of the authors declare that they have no relevant conflicts of interest.

## Supplementary data

Supplementary Tables

Supplementary Checklist
