# Supplementary figures and images for: Associations between dietary intake and asthma outcomes: Evidence from pooled analysis in two independent multiethnic Asian cohorts
Source: J Allergy Clin Immunol Glob. 2026 Jan 20;5(2):100648. doi: 10.1016/j.jacig.2026.100648 (PMC12878681; doi:10.1016/j.jacig.2026.100648)

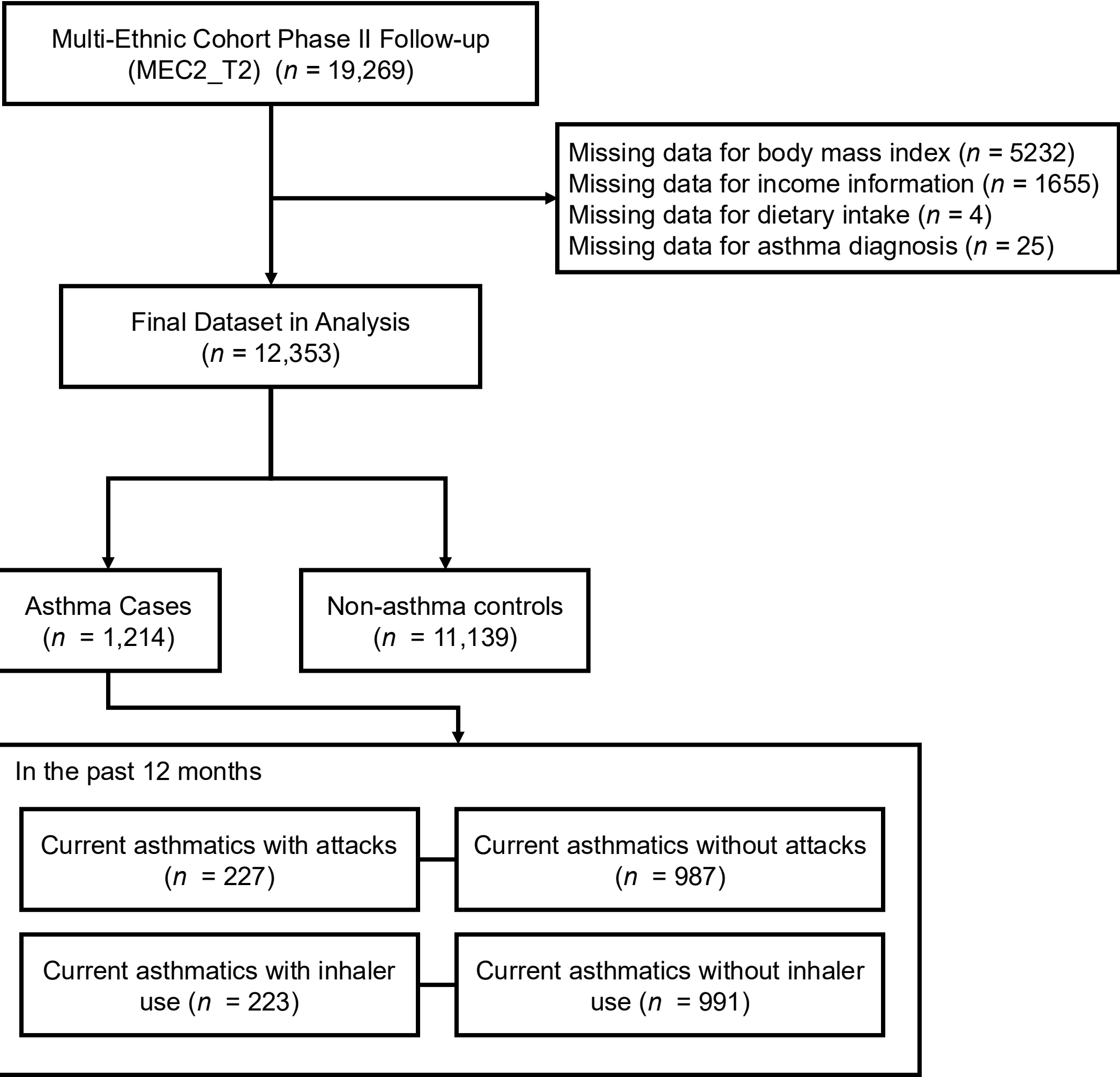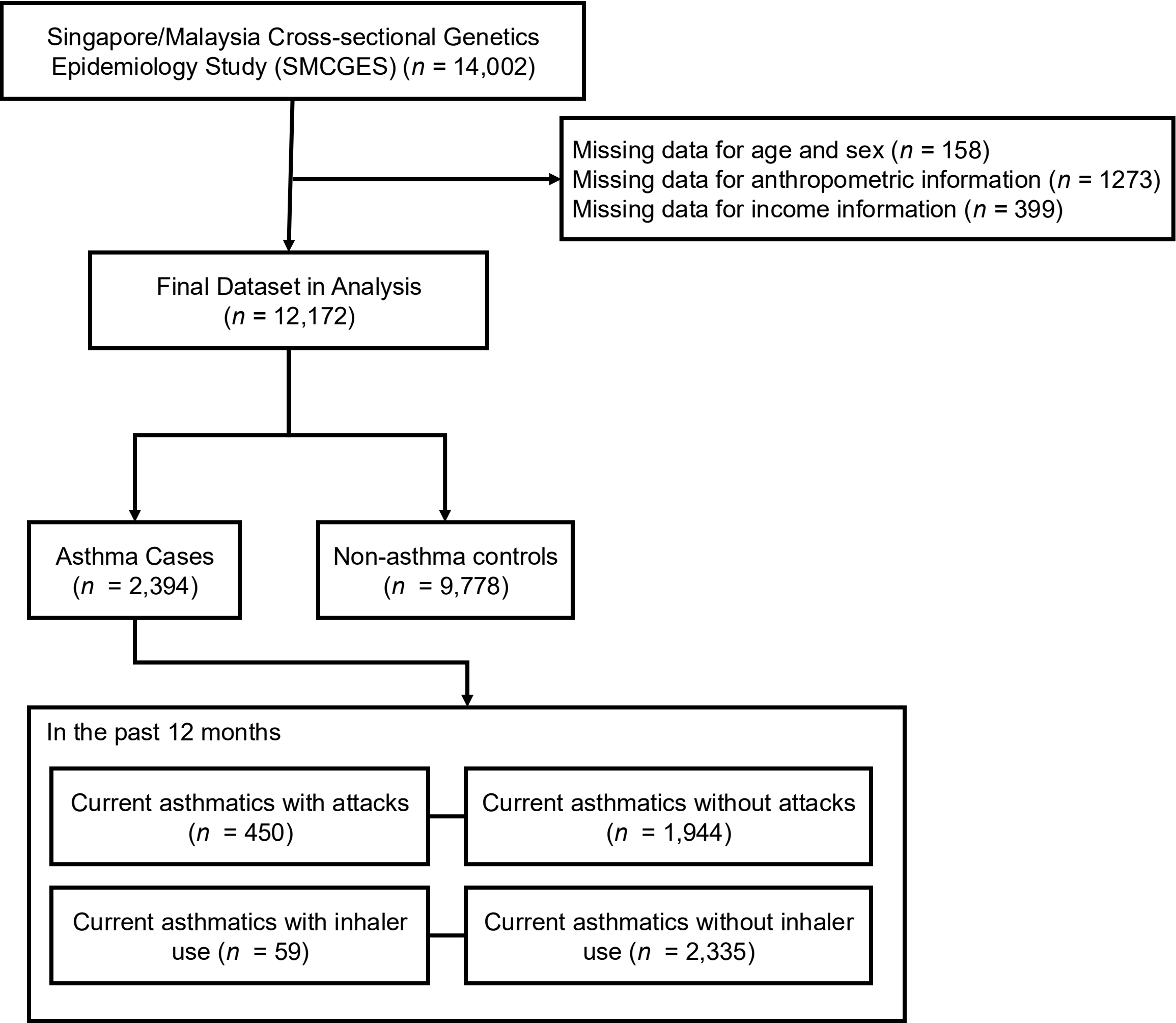

Supplement: Supplementary Figure 1 [file mmc1.pdf]

## MEC2\_T2 (*n* = 12,353)

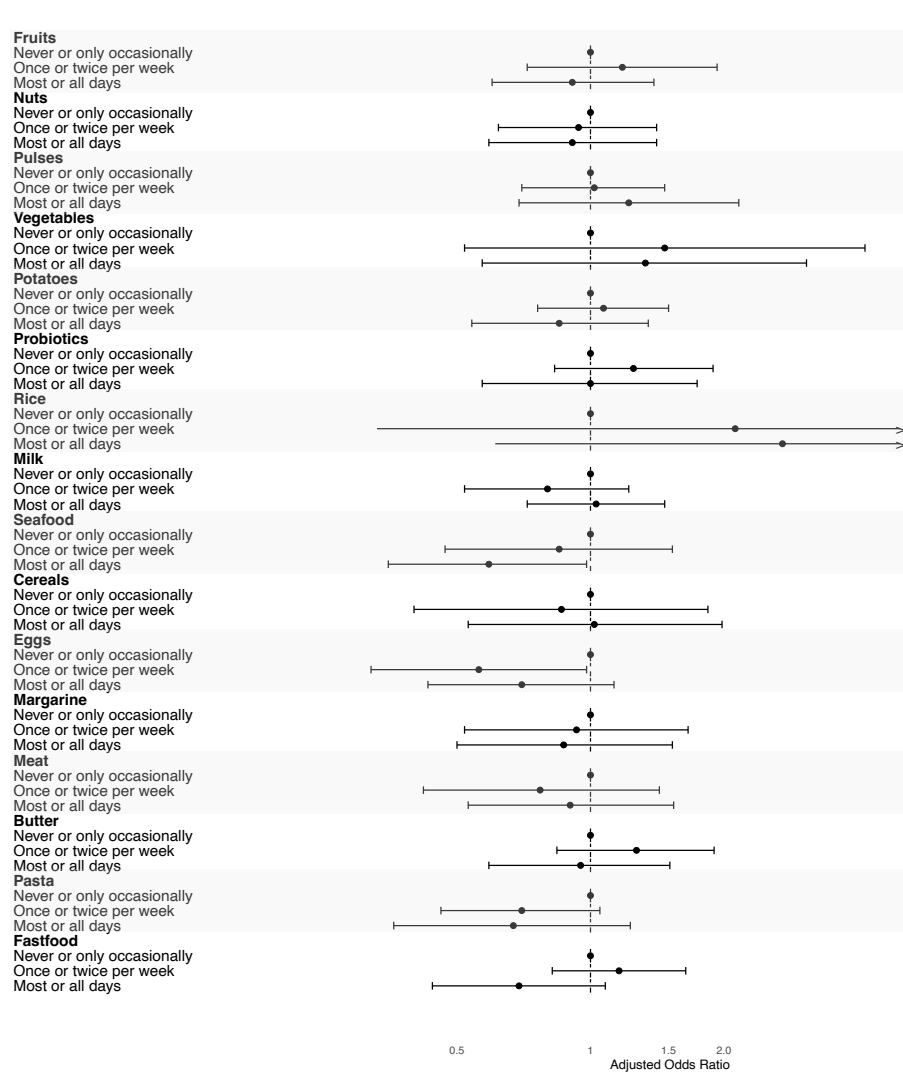

## SMCGES (*n* = 12,172)

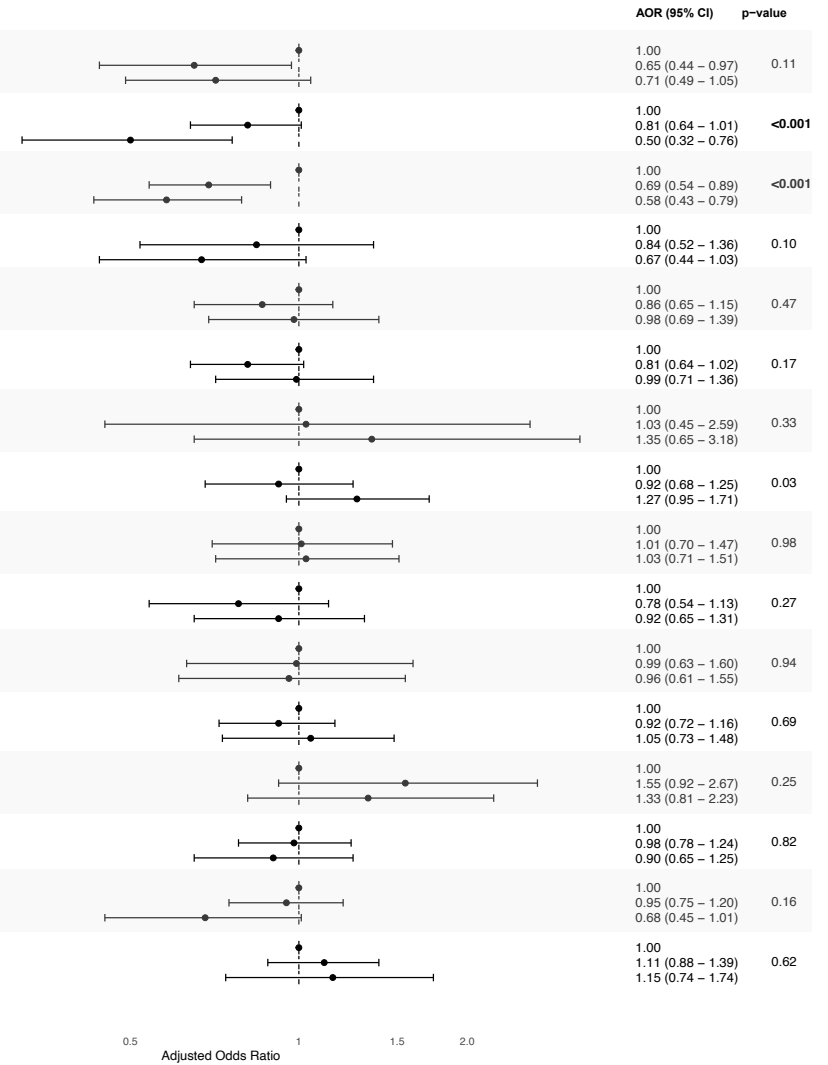

Supplement: Supplementary Figure 2 [file mmc2.pdf]

# MEC2\_T2 (n = 12,353)

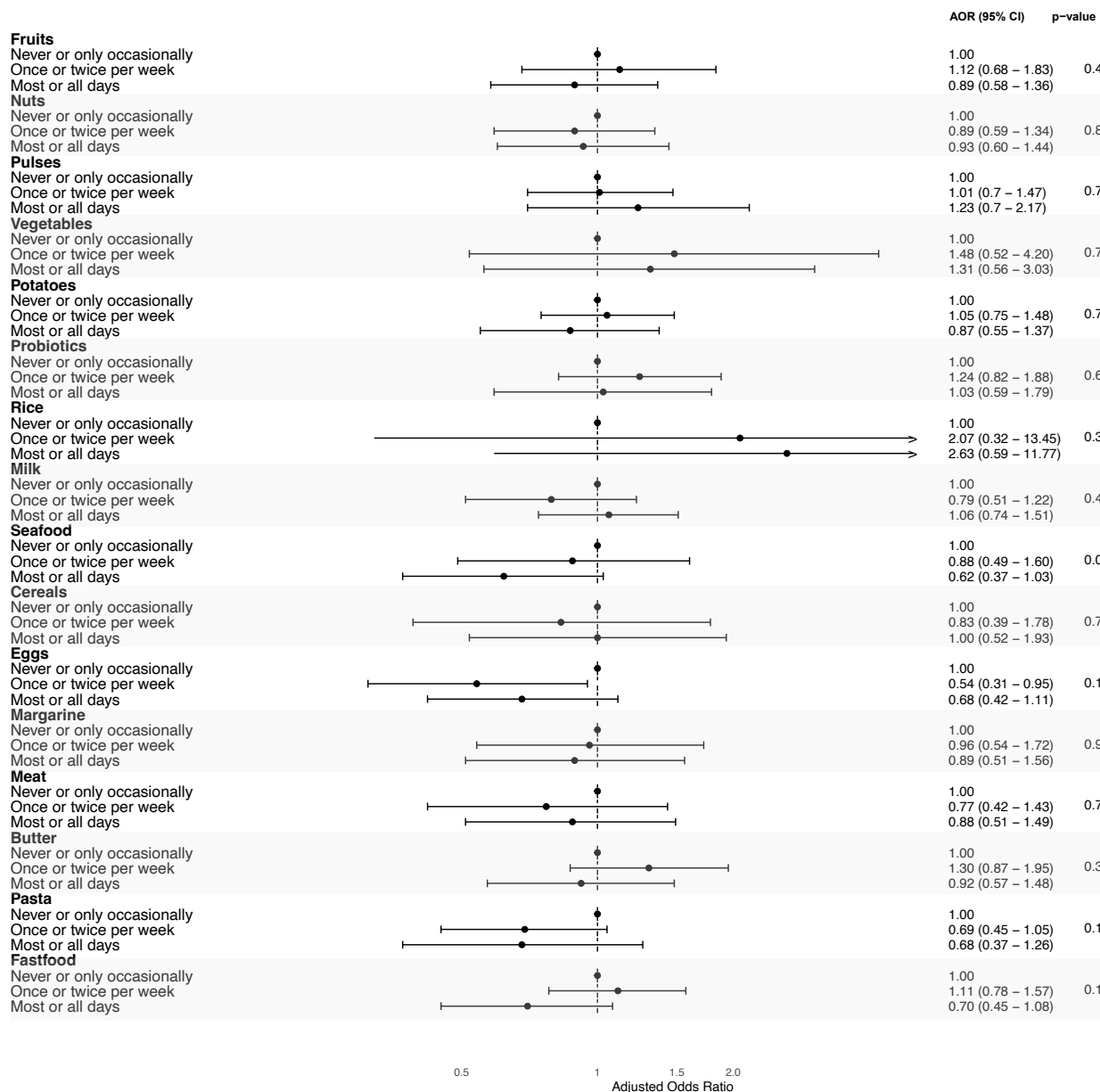

# SMCGES (n = 12,172)

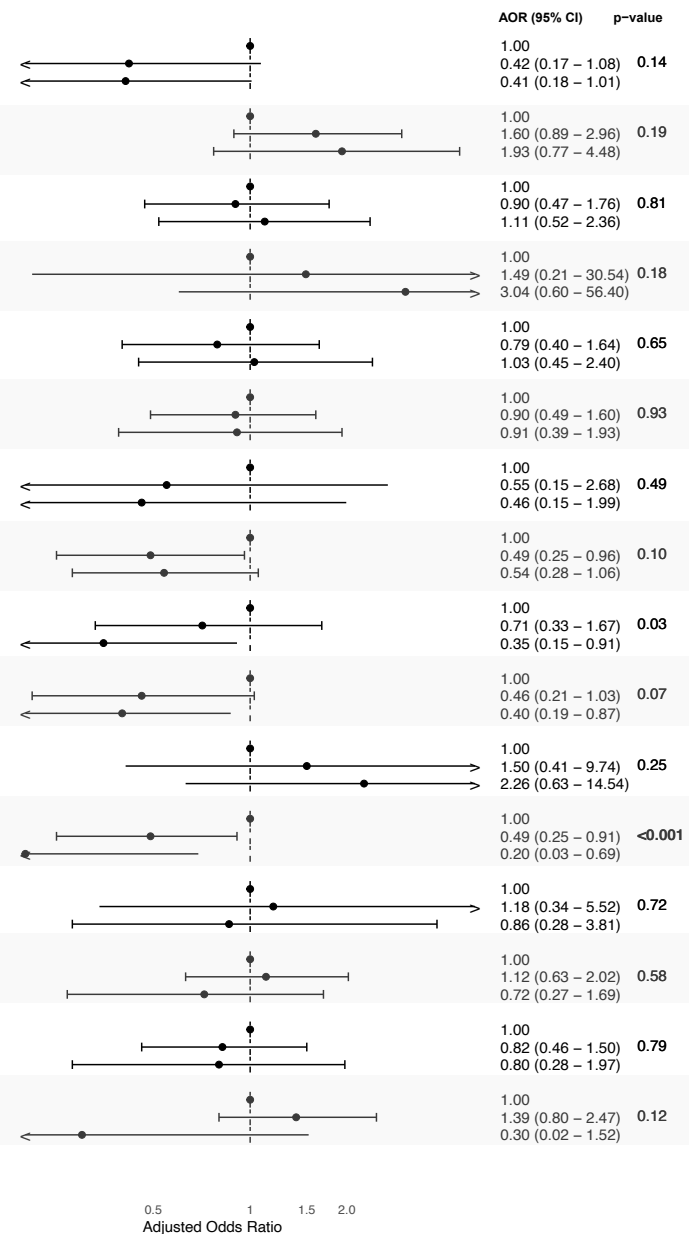

Supplement: Supplementary Figure 3 [file mmc3.pdf]

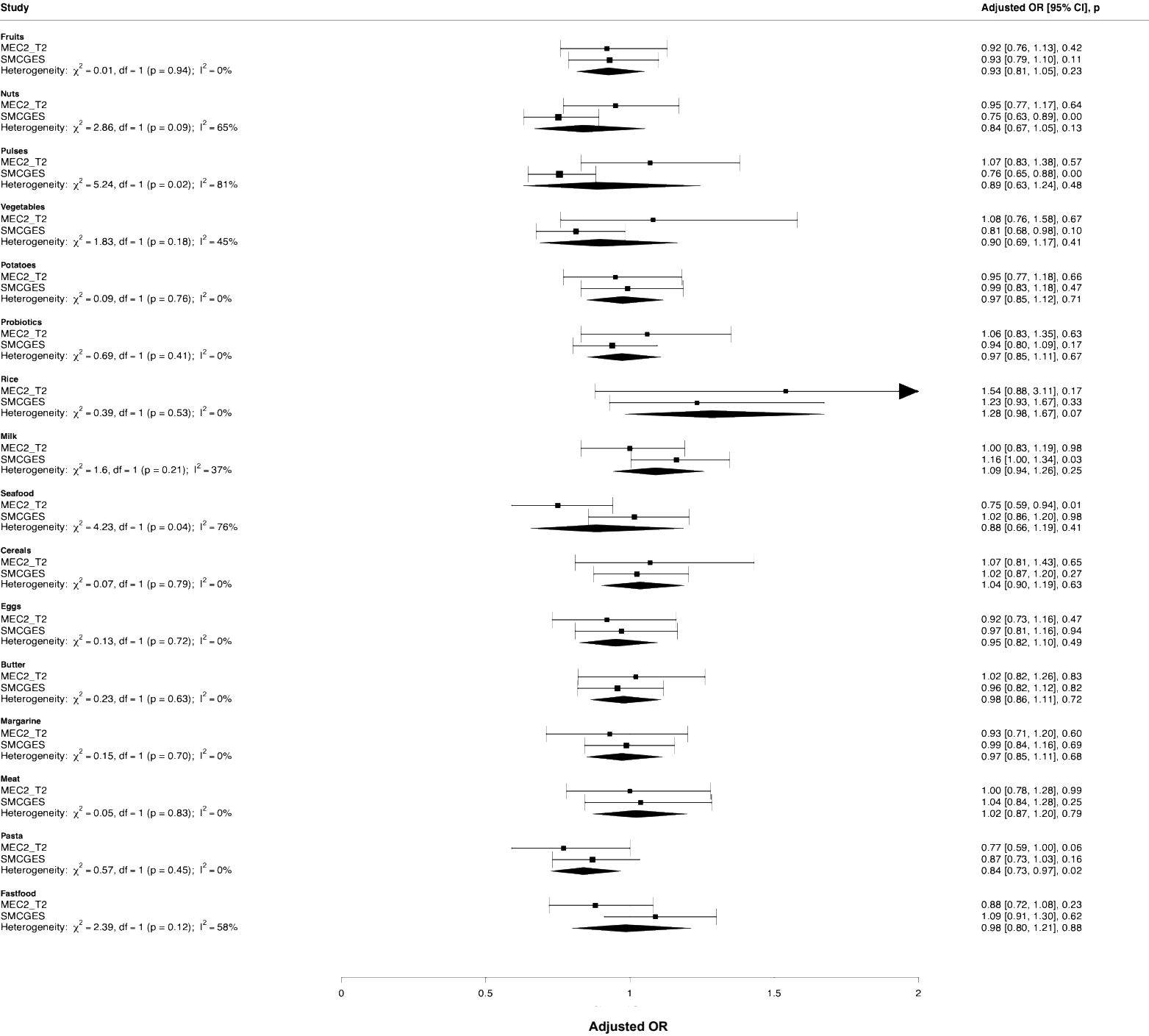

Supplement: Supplementary Figure 4 [file mmc4.pdf]

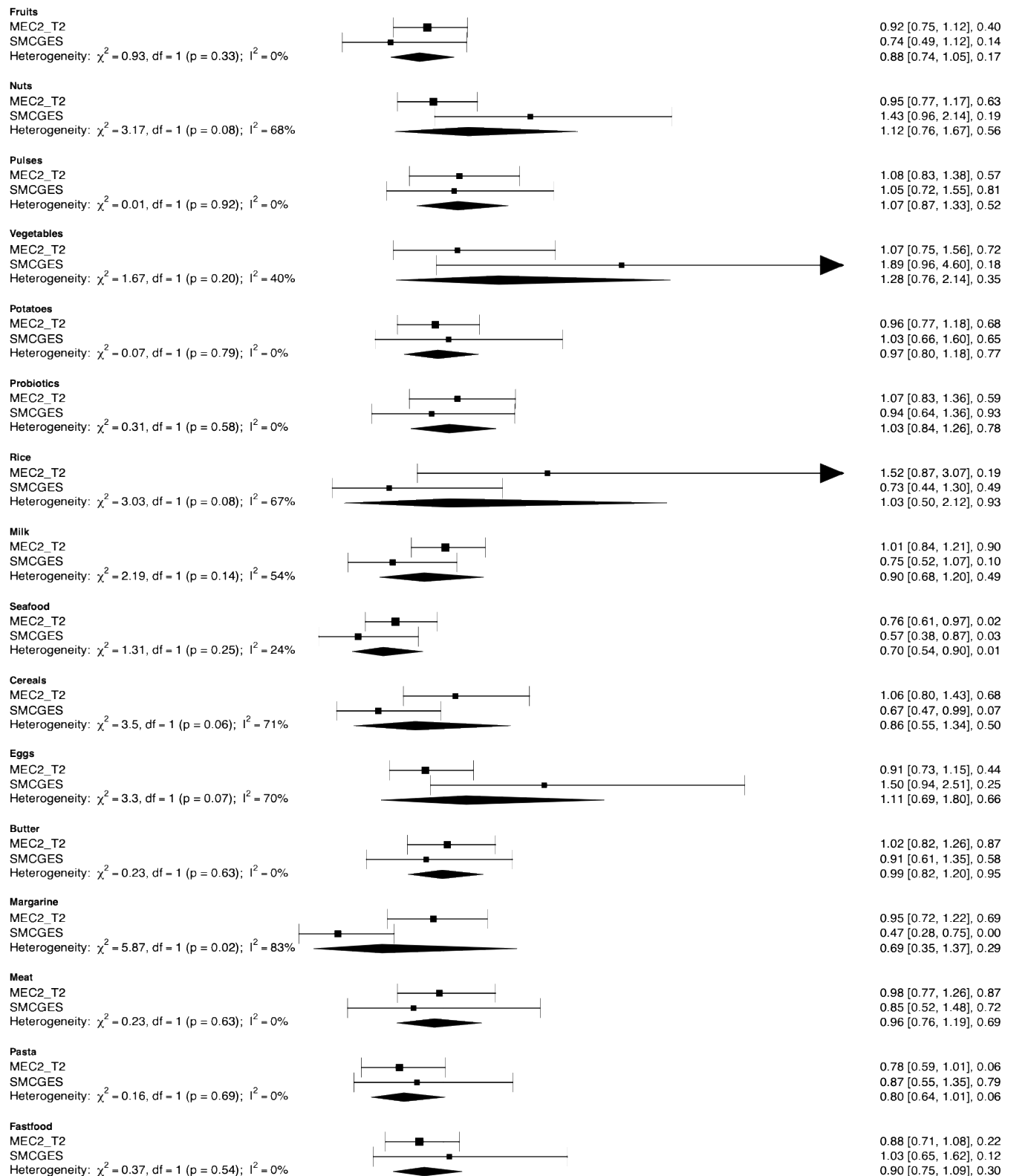

Supplement: Supplementary Figure 5 [file mmc5.pdf]
