# Supplementary material for: Associations between dietary intake and asthma outcomes: Evidence from pooled analysis in two independent multiethnic Asian cohorts
Source: J Allergy Clin Immunol Glob. 2026 Jan 20;5(2):100648. doi: 10.1016/j.jacig.2026.100648 (PMC12878681; doi:10.1016/j.jacig.2026.100648)
Supplement: Supplementary Tables [file mmc6.docx]

**Supplemental Table 1.** List of food items derived from the 163-item semi-quantitative food frequency questionnaire utilized in the Multi-Ethnic Cohort Phase II follow-up (MEC2_T2) cohort.

| **Food Group** | **Number of Food Items Included from the FFQ** | **Food Items** |
| --- | --- | --- |
| Butter | 1 | - Butter |
| Margarine | 1 | - Margarine |
| Nuts | 1 | - Nuts |
| Probiotic drinks | 1 | - Yoghurt drink |
| Potatoes | 2 | - Begedil - Potato |
| Milk | 3 | - Low-fat milk - Non-fat milk - Whole milk |
| Pulses | 4 | - Dhal - Long beans - Peas - Other beans |
| Fast Food | 5 | - Beef burgers (include bread bun) - Deep fried chicken - French fries - Fried onion - Pizza |
| Pasta | 5 | - Pasta dish with meat/fish/seafood/vegetables - Pasta; wholemeal - Tomato sauce (pasta) - Creamy sauce (pasta) - Pasta without sauce, e.g. with oil, seasoning, cheese |
| Eggs | 7 | - Egg; based mixed dishes (containing beancurd) - Egg; based mixed dishes (containing chicken) - Egg; based mixed dishes (containing fish) - Egg; based mixed dishes (containing pork) - Egg; based mixed dishes (containing vegetables) - Egg; boiled/half-boiled/steamed - Egg; fried/scrambled/braised |
| Seafood (including fish) | 17 | - Bivalves, e.g. oysters; cockles; mussels; scallops - Canned fish - Crab - Fish/seafood; boiled/steamed/in soup - Fish/seafood curry made with coconut - Fish/seafood curry made without coconut - Fish/seafood; deep-fried - Fish/seafood; roasted/grilled/baked - Fish/seafood; stewed/braised - Fish/seafood; stir-fried/pan-fried - Oily fish - Prawns - Raw fish/sashimi - Salted fish/dried fish - Squid; cuttlefish and mollusks - Sushi roll/nigiri - White fish |
| Fruit | 17 | - Apple - Avocado - Banana - Blueberries - Dragon fruit - Dried fruit - Durian - Grape - Guava - Kiwi - Mango - Melon - Orange - Papaya - Pear - Pineapple - Strawberries |
| Cereals (including bread) | 19 | - 3-in-1 cereal - Bread; white - Bread; with fruit/nut/seeds - Bread; wholemeal - Breakfast cereal; flavoured; contains wholegrains - Breakfast cereal; flavoured; not wholegrain - Breakfast cereal; mixed; contains wholegrains - Breakfast cereal; mixed; not wholegrain - Breakfast cereal; plain; contains wholegrains - Breakfast cereal; plain; not wholegrain - Chapati - Filled buns; sweet - Filled buns; savoury - French toast - Murtabak - Oats - Puri - Roti prata; plain or with egg/cheese - Thosai; plain or with filling |
| Rice | 20 | - Flavoured rice - Flavoured rice; unpolished - Flavoured rice; white - Flavoured rice dish (mixed with meat/vegetables) - Fried rice; mixed white/unpolished rice - Fried rice; plain or with meat/fish - Fried rice; with vegetables - Fried rice; white rice - Fried rice; unpolished rice - Glutinous rice - Idli - Longtong - Porridge; rice; mixed with white and brown; flavoured - Porridge; rice; mixed white and brown; plain - Porridge; unpolished rice; plain - Porridge; white rice; plain - Rice roll - Rice; mixed white & red/brown - Rice; unpolished - Rice; white |
| Meat | 25 | - Beef; lean - Beef; lean and fat - Chicken; boiled/steamed/in soup - Chicken; roasted/grilled/baked - Chicken; stewed/braised - Chicken; stir-fried/pan-fried - Chicken curry made with coconut - Chicken curry without coconut - Chicken/turkey ham - Chicken; with skin - Chicken; without skin - Cured pork products - Meat; boiled/steamed/in soup - Meat; deep-fried - Meat curry made with coconut - Meat curry without coconut - Meat; roasted/grilled/baked - Meat; stewed/braised - Meat; stir-fried/pan-fried - Mutton and lamb; lean - Mutton and lamb; lean and fat - Organ meat - Pork; lean - Pork; lean and fat - Processed chicken products |
| Vegetables (including roots and green) | 25 | - Beansprouts - Broccoli - Cabbage - Capsicum - Carrot - Cauliflowers - Celery - Coleslaw - Corn - Cucumber - Eggplant - Gourd - Kailan - Kang kong - Lettuce - Lotus root - Mushrooms - Mustard greens (chye sim) - Okra - Pumpkin - Raita - Seaweed - Spinach - Tomato; raw - Yam |

For more information on the validated 163-item FFQ, please refer to the Whitton C *et al*., 2017 (doi:10.3390/nu9101059)

**Supplemental Table 2**. Intake frequency of the 16 food groups among individuals from the multi-ethnic cohort phase II follow-up (MEC2_T2) cohort (*n* = 12,353) and Singapore/Malaysia Cross-sectional Genetics Epidemiology Study (SMCGES) cohort (*n* = 12,353) between non-asthmatic controls and asthma cases.

| Dietary Intake | **MEC2_T2 (*n* = 12,353)** | | | **SMCGES (*n* = 12,172)** | | |
| --- | --- | --- | --- | --- | --- | --- |
|  | Non-asthmatic controls  (*n* = 11,139) | Asthma cases^1^  (*n* = 1,214) | P-value | Non-asthmatic controls  (*n* = 9,777) | Asthma cases^1^  (*n* = 2,394) | P-value |
| **Fruits** | | | | | | |
| - Never or only occasionally | 1,210 (10.9) | 198 (16.3) | 6.68 x 10^-9^ | 441 (4.5) | 183 (7.6) | 2.95 x 10^-9^ |
| - Once or twice per week | 2,138 (19.2) | 252 (20.8) |  | 3,366 (34.4) | 814 (34.0) |  |
| - Most or all days | 7,791 (69.9) | 764 (62.9) |  | 5,971 (61.1) | 1,397 (58.4) |  |
| **Nuts** | | | | | | |
| - Never or only occasionally | 6,707 (60.2) | 779 (64.2) | 9.71 x 10^-1^ | 4,091 (41.8) | 1,066 (44.5) | 3.78 x 10^-2^ |
| - Once or twice per week | 2,354 (21.1) | 243 (20.0) |  | 4,825 (49.3) | 1,113 (46.5) |  |
| - Most or all days | 2,078 (18.7) | 192 (15.8) |  | 862 (8.8) | 215 (9.0) |  |
| **Pulses (peas, beans, lentils)** | | | | | | |
| - Never or only occasionally | 7,100 (63.7) | 816 (67.2) | 1.79 x 10^-1^ | 2,106 (21.5) | 644 (26.9) | 6.90 x 10^-11^ |
| - Once or twice per week | 2,978 (26.7) | 307 (25.3) |  | 5,660 (57.9) | 1,209 (50.5) |  |
| - Most or all days | 1,061 (9.5) | 91 (7.5) |  | 2,012 (20.6) | 541 (22.6) |  |
| **Vegetables (roots and green)** | | | | | | |
| - Never or only occasionally | 331 (3.0) | 38 (3.1) | 3.41 x 10^-1^ | 259 (2.6) | 135 (5.6) | 4.89 x 10^-13^ |
| - Once or twice per week | 380 (3.4) | 51 (4.2) |  | 1,234 (12.6) | 317 (13.2) |  |
| - Most or all days | 10,428 (93.6) | 1,125 (92.7) |  | 8,285 (84.7) | 1,942 (81.1) |  |
| **Potatoes** | | | | | | |
| - Never or only occasionally | 5,958 (53.5) | 633 (52.1) | 6.02 x 10^-1^ | 1,626 (16.6) | 425 (17.8) | 6.94 x 10^-2^ |
| - Once or twice per week | 3,677 (33.0) | 407 (33.5) |  | 6,564 (67.1) | 1,542 (64.4) |  |
| - Most or all days | 1,504 (13.5) | 174 (14.3) |  | 1,588 (16.2) | 427 (17.8) |  |
| **Probiotic Drinks** | | | | | | |
| - Never or only occasionally | 8,652 (77.7) | 930 (76.6) | 1.59 x 10^-2^ | 4,107 (42.0) | 1,118 (46.7) | 3.81 x 10^-2^ |
| - Once or twice per week | 1,592 (14.3) | 177 (14.6) |  | 4,243 (43.4) | 951 (39.7) |  |
| - Most or all days | 895 (8.0) | 107 (8.8) |  | 1,428 (14.6) | 325 (13.6) |  |
| **Rice** | | | | | | |
| - Never or only occasionally | 175 (1.6) | 23 (1.9) | 6.92 x 10^-1^ | 241 (2.5) | 53 (2.2) | 3.81 x 10^-1^ |
| - Once or twice per week | 207 (1.9) | 23 (1.9) |  | 1,013 (10.4) | 229 (9.6) |  |
| - Most or all days | 10,757 (96.6) | 1,168 (96.2) |  | 8,524 (87.2) | 2,112 (88.2) |  |
| **Milk** | | | | | | |
| - Never or only occasionally | 6,622 (59.4) | 689 (56.8) | 1.79 x 10^-1^ | 1,900 (19.4) | 506 (21.1) | 4.27 x 10^-3^ |
| - Once or twice per week | 1,811 (16.3) | 215 (17.7) |  | 4,360 (44.6) | 979 (40.9) |  |
| - Most or all days | 2,706 (24.3) | 310 (25.5) |  | 3,518 (36.0) | 909 (38.0) |  |
| **Seafood (including fish)** | | | | | | |
| - Never or only occasionally | 1,272 (11.4) | 97 (8.0) | 8.06 x 10^-4^ | 1,024 (10.5) | 217 (9.1) | 7.44 x 10^-4^ |
| - Once or twice per week | 1,582 (14.2) | 193 (15.9) |  | 4,932 (50.4) | 1,144 (47.8) |  |
| - Most or all days | 8,285 (74.4) | 924 (76.1) |  | 3,822 (39.1) | 1,033 (43.1) |  |
| **Cereals (including bread)** | | | | | | |
| - Never or only occasionally | 692 (6.2) | 76 (6.3) | 6.94 x 10^-2^ | 974 (10.0) | 240 (10.0) | 4.61 x 10^-3^ |
| - Once or twice per week | 1,437 (12.9) | 185 (15.2) |  | 3,776 (38.6) | 840 (35.1) |  |
| - Most or all days | 9,010 (80.9) | 953 (78.5) |  | 5,028 (51.4) | 1,314 (54.9) |  |
| **Eggs** | | | | | | |
| - Never or only occasionally | 1,117 (10.0) | 106 (8.7) | 1.84 x 10^-1^ | 465 (4.8) | 121 (5.1) | 6.53 x 10^-1^ |
| - Once or twice per week | 2,342 (21.0) | 242 (19.9) |  | 4,043 (41.3) | 1,005 (42.0) |  |
| - Most or all days | 7,680 (68.9) | 866 (71.3) |  | 5,270 (53.9) | 1,268 (53.0) |  |
| **Margarine** | | | | | | |
| - Never or only occasionally | 9,413 (84.5) | 1,024 (84.3) | 9.71 x 10^-1^ | 5,559 (56.9) | 1,270 (53.0) | 5.93 x 10^-5^ |
| - Once or twice per week | 823 (7.4) | 92 (7.6) |  | 3,447 (35.3) | 877 (36.6) |  |
| - Most or all days | 903 (8.1) | 98 (8.1) |  | 772 (7.9) | 247 (10.3) |  |
| **Meat** | | | | | | |
| - Never or only occasionally | 1,178 (10.6) | 97 (8.0) | 8.90 x 10^-3^ | 429 (4.4) | 106 (4.4) | 1.26 x 10^-2^ |
| - Once or twice per week | 1,733 (15.6) | 178 (14.7) |  | 1,074 (11.0) | 314 (13.1) |  |
| - Most or all days | 8,228 (73.9) | 939 (77.3) |  | 8,275 (84.6) | 1,974 (82.5) |  |
| **Butter** | | | | | | |
| - Never or only occasionally | 8,086 (72.6) | 880 (72.5) | 4.90 x 10^-1^ | 4,174 (42.7) | 935 (39.1) | 4.11 x 10^-4^ |
| - Once or twice per week | 1,614 (14.5) | 188 (15.5) |  | 4,408 (45.1) | 1,108 (46.3) |  |
| - Most or all days | 1,439 (12.9) | 146 (12.0) |  | 1,196 (12.2) | 351 (14.7) |  |
| **Pasta** | | | | | | |
| - Never or only occasionally | 8,739 (78.5) | 864 (71.2) | 2.25 x 10^-8^ | 3,649 (37.3) | 897 (37.5) | 3.97 x 10^-1^ |
| - Once or twice per week | 1,757 (15.8) | 246 (20.3) |  | 5,125 (52.4) | 1,230 (51.4) |  |
| - Most or all days | 643 (5.8) | 104 (8.6) |  | 1,004 (10.3) | 267 (11.2) |  |
| **Fast Food** | | | | | | |
| - Never or only occasionally | 6,607 (59.3) | 570 (47.0) | 2.07 x 10^-2^ | 3,709 (37.9) | 925 (38.6) | 2.40 x 10^-1^ |
| - Once or twice per week | 3,234 (29.0) | 396 (32.6) |  | 5,428 (55.5) | 1,293 (54.0) |  |
| - Most or all days | 1,298 (11.7) | 248 (20.4) |  | 641 (6.6) | 176 (7.4) |  |

**Supplemental Table 3**. Intake frequency of the 16 food groups among individuals from the multi-ethnic cohort phase II follow-up (MEC2_T2) cohort (*n* = 12,353) and Singapore/Malaysia Cross-sectional Genetics Epidemiology Study (SMCGES) cohort (*n* = 12,353) between asthmatics with recent attacks and those without.

| Dietary Intake | **MEC2_T2 (*n* = 12,353)** | | | **SMCGES (*n* = 12,172)** | | |
| --- | --- | --- | --- | --- | --- | --- |
|  | Asthmatics without recent attacks  (*n* = 987) | Asthmatics with recent attacks  (*n* = 227) | P-value | Asthmatics without recent attacks  (*n* = 1932) | Asthmatics with recent attacks  (*n* = 444) | P-value |
| **Fruits** | | | | | | |
| - Never or only occasionally | 159 (16.1) | 39 (17.2) | 3.60 x 10^-1^ | 127 (6.5) | 56 (12.4) | 1.15 x 10^-4^ |
| - Once or twice per week | 198 (20.1) | 54 (23.8) |  | 671 (34.5) | 143 (31.8) |  |
| - Most or all days | 630 (63.8) | 134 (59.0) |  | 1,146 (59.0) | 251 (55.8) |  |
| **Nuts** | | | | | | |
| - Never or only occasionally | 624 (63.2) | 155 (68.3) | 3.57 x 10^-1^ | 830 (42.7) | 236 (52.4) | 4.96 x 10^-4^ |
| - Once or twice per week | 203 (20.6) | 40 (17.6) |  | 928 (47.7) | 185 (41.1) |  |
| - Most or all days | 160 (16.2) | 32 (14.1) |  | 186 (9.6) | 29 (6.4) |  |
| **Pulses (peas, beans, lentils)** | | | | | | |
| - Never or only occasionally | 667 (67.6) | 149 (65.6) | 3.79 x 10^-1^ | 485 (24.9) | 159 (35.3) | 4.10 x 10^-5^ |
| - Once or twice per week | 251 (25.4) | 56 (24.7) |  | 1,011 (52.0) | 198 (44.0) |  |
| - Most or all days | 69 (7.0) | 22 (9.7) |  | 448 (23.0) | 93 (20.7) |  |
| **Vegetables (roots and green)** | | | | | | |
| - Never or only occasionally | 29 (2.9) | 9 (4.0) | 1.80 x 10^-1^ | 90 (4.6) | 45 (10.0) | 6.04 x 10^-7^ |
| - Once or twice per week | 37 (3.7) | 14 (6.2) |  | 241 (12.4) | 76 (16.9) |  |
| - Most or all days | 921 (93.3) | 204 (89.9) |  | 1,613 (83.0) | 329 (73.1) |  |
| **Potatoes** | | | | | | |
| - Never or only occasionally | 521 (52.8) | 112 (49.3) | 6.09 x 10^-1^ | 333 (17.1) | 92 (20.4) | 2.50 x 10^-2^ |
| - Once or twice per week | 325 (32.9) | 82 (36.1) |  | 1,277 (65.7) | 265 (58.9) |  |
| - Most or all days | 141 (14.3) | 33 (14.5) |  | 334 (17.2) | 93 (20.7) |  |
| **Probiotic Drinks** | | | | | | |
| - Never or only occasionally | 763 (77.3) | 167 (73.6) | 2.57 x 10^-1^ | 881 (45.3) | 237 (52.7) | 8.44 x 10^-3^ |
| - Once or twice per week | 136 (13.8) | 41 (18.1) |  | 800 (41.2) | 151 (33.6) |  |
| - Most or all days | 88 (8.9) | 19 (8.4) |  | 263 (13.5) | 62 (13.8) |  |
| **Rice** | | | | | | |
| - Never or only occasionally | 21 (2.1) | 2 (0.9) | 4.54 x 10^-1^ | 45 (2.3) | 8 (1.8) | 1.49 x 10^-1^ |
| - Once or twice per week | 19 (1.9) | 4 (1.8) |  | 196 (10.1) | 33 (7.3) |  |
| - Most or all days | 947 (95.9) | 221 (97.4) |  | 1,703 (87.6) | 409 (90.9) |  |
| **Milk** | | | | | | |
| - Never or only occasionally | 563 (57.0) | 126 (55.5) | 5.59 x 10^-1^ | 418 (21.5) | 88 (19.6) | 9.35 x 10^-5^ |
| - Once or twice per week | 178 (18.0) | 37 (16.3) |  | 827 (42.5) | 152 (33.8) |  |
| - Most or all days | 246 (24.9) | 64 (28.2) |  | 699 (36.0) | 210 (46.7) |  |
| **Seafood (including fish)** | | | | | | |
| - Never or only occasionally | 71 (7.2) | 26 (11.5) | 7.49 x 10^-3^ | 162 (8.3) | 55 (12.2) | 2.05 x 10^-2^ |
| - Once or twice per week | 147 (14.9) | 46 (20.3) |  | 927 (47.7) | 217 (48.2) |  |
| - Most or all days | 769 (77.9) | 155 (68.3) |  | 855 (44.0) | 178 (39.6) |  |
| **Cereals (including bread)** | | | | | | |
| - Never or only occasionally | 63 (6.4) | 13 (5.7) | 4.59 x 10^-1^ | 185 (9.5) | 55 (12.2) | 3.55 x 10^-2^ |
| - Once or twice per week | 156 (15.8) | 29 (12.8) |  | 703 (36.2) | 137 (30.4) |  |
| - Most or all days | 768 (77.8) | 185 (81.5) |  | 1,056 (54.3) | 258 (57.3) |  |
| **Eggs** | | | | | | |
| - Never or only occasionally | 76 (7.7) | 30 (13.2) | 2.24 x 10^-2^ | 90 (4.6) | 31 (6.9) | 1.47 x 10^-2^ |
| - Once or twice per week | 196 (19.9) | 47 (20.7) |  | 800 (41.2) | 205 (45.6) |  |
| - Most or all days | 7716 (781.8) | 150 (66.1) |  | 1,054 (54.2) | 214 (47.6) |  |
| **Margarine** | | | | | | |
| - Never or only occasionally | 832 (84.3) | 192 (84.6) | 9.94 x 10^-1^ | 1,024 (52.7) | 246 (54.7) | 1.29 x 10^-2^ |
| - Once or twice per week | 75 (7.6) | 17 (7.5) |  | 728 (37.4) | 149 (33.1) |  |
| - Most or all days | 80 (8.1) | 18 (7.9) |  | 192 (9.9) | 55 (12.2) |  |
| **Meat** | | | | | | |
| - Never or only occasionally | 73 (7.4) | 24 (10.6) | 1.40 x 10^-1^ | 80 (4.1) | 26 (5.8) | 1.31 x 10^-7^ |
| - Once or twice per week | 140 (14.2) | 38 (16.7) |  | 221 (11.4) | 93 (20.7) |  |
| - Most or all days | 774 (78.4) | 165 (72.7) |  | 1,643 (84.5) | 331 (73.6) |  |
| **Butter** | | | | | | |
| - Never or only occasionally | 724 (73.4) | 156 (68.7) | 2.56 x 10^-1^ | 754 (38.8) | 181 (40.2) | 7.47 x 10^-1^ |
| - Once or twice per week | 145 (14.7) | 43 (18.9) |  | 907 (46.7) | 201 (44.7) |  |
| - Most or all days | 118 (12.0) | 28 (12.3) |  | 283 (14.6) | 68 (15.1) |  |
| **Pasta** | | | | | | |
| - Never or only occasionally | 687 (69.6) | 177 (78.0) | 4.15 x 10^-2^ | 699 (36.0) | 198 (44.0) | 1.50 x 10^-3^ |
| - Once or twice per week | 210 (21.3) | 36 (15.9) |  | 1,013 (52.1) | 217 (48.2) |  |
| - Most or all days | 90 (9.1) | 14 (6.2) |  | 232 (11.9) | 35 (7.8) |  |
| **Fast Food** | | | | | | |
| - Never or only occasionally | 459 (46.5) | 111 (48.9) | 2.27 x 10^-1^ | 753 (38.7) | 172 (38.2) | 7.34 x 10^-1^ |
| - Once or twice per week | 317 (32.1) | 79 (34.8) |  | 1,052 (54.1) | 241 (53.6) |  |
| - Most or all days | 211 (21.4) | 37 (16.3) |  | 139 (7.2) | 37 (8.2) |  |

**Supplemental Table 4**. Intake frequency of the 16 food groups among individuals from the multi-ethnic cohort phase II follow-up (MEC2_T2) cohort (*n* = 12,353) and Singapore/Malaysia Cross-sectional Genetics Epidemiology Study (SMCGES) cohort (*n* = 12,353) between asthmatics with recent attacks and those without.

| Dietary Intake | **MEC2_T2 (*n* = 12,353)** | | | **SMCGES (*n* = 12,172)** | | |
| --- | --- | --- | --- | --- | --- | --- |
|  | Asthmatics without recent use of inhalers  (*n* = 991) | Asthmatics with recent use of inhalers  (*n* = 223) | P-value | Asthmatics without recent use of inhalers  (*n* = 2335) | Asthmatics with recent use of inhalers  (*n* = 59) | P-value |
| **Fruits** | | | | | | |
| - Never or only occasionally | 159 (16.0) | 39 (17.5) | 4.27 x 10^-1^ | 175 (7.5) | 8 (13.6) | 5.03 x 10^-1^ |
| - Once or twice per week | 200 (20.2) | 52 (23.3) |  | 796 (34.1) | 18 (30.5) |  |
| - Most or all days | 632 (63.8) | 132 (59.2) |  | 1364 (58.4) | 33 (55.9) |  |
| **Nuts** | | | | | | |
| - Never or only occasionally | 626 (63.2) | 153 (68.6) | 2.98 x 10^-1^ | 1047 (44.8) | 19 (32.2) | 1.19 x 10^-1^ |
| - Once or twice per week | 205 (20.7) | 38 (17.0) |  | 1081 (46.3) | 32 (54.2) |  |
| - Most or all days | 160 (16.1) | 32 (14.3) |  | 207 (8.9) | 8 (13.6) |  |
| **Pulses (peas, beans, lentils)** | | | | | | |
| - Never or only occasionally | 670 (67.6) | 146 (65.5) | 3.31 x 10^-1^ | 628 (26.9) | 16 (27.1) | 6.65 x 10^-1^ |
| - Once or twice per week | 252 (25.4) | 55 (24.7) |  | 1182 (50.6) | 27 (45.8) |  |
| - Most or all days | 69 (7.0) | 22 (9.9) |  | 525 (22.5) | 16 (27.1) |  |
| **Vegetables (roots and green)** | | | | | | |
| - Never or only occasionally | 29 (2.9) | 9 (4.0) | 1.51 x 10^-1^ | 134 (5.7) | 1 (1.7) | 1.56 x 10^-1^ |
| - Once or twice per week | 37 (3.7) | 14 (6.3) |  | 313 (13.4) | 4 (6.8) |  |
| - Most or all days | 925 (93.3) | 200 (89.7) |  | 1888 (80.9) | 54 (91.5) |  |
| **Potatoes** | | | | | | |
| - Never or only occasionally | 523 (52.8) | 110 (49.3) | 6.36 x 10^-1^ | 413 (17.7) | 12 (20.3) | 5.33 x 10^-1^ |
| - Once or twice per week | 327 (33.0) | 80 (35.9) |  | 1508 (64.6) | 34 (57.6) |  |
| - Most or all days | 141 (14.2) | 33 (14.8) |  | 414 (17.7) | 13 (22.0) |  |
| **Probiotic Drinks** | | | | | | |
| - Never or only occasionally | 766 (77.3) | 164 (73.5) | 2.90 x 10^-1^ | 1089 (46.6) | 29 (49.2) | 7.93 x 10^-1^ |
| - Once or twice per week | 137 (13.8) | 40 (17.9) |  | 930 (39.8) | 21 (35.6) |  |
| - Most or all days | 88 (8.9) | 19 (8.5) |  | 316 (13.5) | 9 (15.3) |  |
| **Rice** | | | | | | |
| - Never or only occasionally | 21 (2.1) | 2 (0.9) | 4.76 x 10^-1^ | 50 (2.1) | 3 (5.1) | 6.39 x 10^-2^ |
| - Once or twice per week | 19 (1.9) | 4 (1.8) |  | 220 (9.4) | 9 (15.3) |  |
| - Most or all days | 951 (96.0) | 217 (97.3) |  | 2065 (88.4) | 47 (79.7) |  |
| **Milk** | | | | | | |
| - Never or only occasionally | 566 (57.1) | 123 (55.2) | 4.56 x 10^-1^ | 488 (20.9) | 18 (30.5) | 1.62 x 10^-1^ |
| - Once or twice per week | 179 (18.1) | 36 (16.1) |  | 960 (41.1) | 19 (32.2) |  |
| - Most or all days | 246 (24.8) | 64 (28.7) |  | 887 (38.0) | 22 (37.3) |  |
| **Seafood (including fish)** | | | | | | |
| - Never or only occasionally | 72 (7.3) | 25 (11.2) | 1.28 x 10^-2^ | 208 (8.9) | 9 (15.3) | 2.49 x 10^-2^ |
| - Once or twice per week | 148 (14.9) | 45 (20.2) |  | 1110 (47.5) | 34 (57.6) |  |
| - Most or all days | 771 (77.8) | 153 (68.6) |  | 1017 (43.6) | 16 (27.1) |  |
| **Cereals (including bread)** | | | | | | |
| - Never or only occasionally | 63 (6.4) | 13 (5.8) | 4.26 x 10^-1^ | 229 (9.8) | 11 (18.6) | 7.75 x 10^-2^ |
| - Once or twice per week | 157 (15.8) | 28 (12.6) |  | 820 (35.1) | 20 (33.9) |  |
| - Most or all days | 771 (77.8) | 182 (81.6) |  | 1286 (55.1) | 28 (47.5) |  |
| **Eggs** | | | | | | |
| - Never or only occasionally | 76 (7.7) | 30 (13.5) | 1.69 x 10^-2^ | 119 (5.1) | 2 (3.4) | 3.38 x 10^-2^ |
| - Once or twice per week | 196 (19.8) | 46 (20.6) |  | 985 (42.2) | 20 (33.9) |  |
| - Most or all days | 719 (72.6) | 147 (65.9) |  | 1231 (52.7) | 37 (62.7) |  |
| **Margarine** | | | | | | |
| - Never or only occasionally | 836 (84.4) | 188 (84.3) | 9.99 x 10^-1^ | 1226 (52.5) | 44 (74.6) | 3.00 x 10^-3^ |
| - Once or twice per week | 75 (7.6) | 17 (7.6) |  | 864 (37.0) | 13 (22.0) |  |
| - Most or all days | 80 (8.1) | 18 (8.1) |  | 245 (10.5) | 2 (3.4) |  |
| **Meat** | | | | | | |
| - Never or only occasionally | 73 (7.4) | 24 (10.8) | 9.94 x 10^-2^ | 103 (4.4) | 3 (5.1) | 5.03 x 10^-1^ |
| - Once or twice per week | 140 (14.1) | 38 (17.0) |  | 304 (13.0) | 10 (16.9) |  |
| - Most or all days | 778 (78.5) | 161 (72.2) |  | 1928 (82.6) | 46 (78.0) |  |
| **Butter** | | | | | | |
| - Never or only occasionally | 727 (73.4) | 153 (68.6) | 2.11 x 10^-1^ | 914 (39.1) | 21 (35.6) | 6.00 x 10^-1^ |
| - Once or twice per week | 145 (14.6) | 43 (19.3) |  | 1077 (46.1) | 31 (52.5) |  |
| - Most or all days | 119 (12.0) | 27 (12.1) |  | 344 (14.7) | 7 (11.9) |  |
| **Pasta** | | | | | | |
| - Never or only occasionally | 690 (69.6) | 174 (78.0) | 4.31 x 10^-2^ | 875 (37.5) | 22 (37.3) | 9.66 x 10^-1^ |
| - Once or twice per week | 211 (21.3) | 35 (15.7) |  | 1199 (51.3) | 31 (52.5) |  |
| - Most or all days | 90 (9.1) | 14 (6.3) |  | 261 (11.2) | 6 (10.2) |  |
| **Fast Food** | | | | | | |
| - Never or only occasionally | 460 (46.4) | 110 (49.3) | 2.90 x 10^-1^ | 904 (38.7) | 21 (35.6) | 1.69 x 10^-1^ |
| - Once or twice per week | 320 (32.3) | 76 (34.1) |  | 1256 (53.8) | 37 (62.7) |  |
| - Most or all days | 211 (21.3) | 37 (16.6) |  | 175 (7.5) | 1 (1.7) |  |
