# Supplementary material for: Associations between dietary intake and asthma outcomes: Evidence from pooled analysis in two independent multiethnic Asian cohorts
Source: J Allergy Clin Immunol Glob. 2026 Jan 20;5(2):100648. doi: 10.1016/j.jacig.2026.100648 (PMC12878681; doi:10.1016/j.jacig.2026.100648)
Supplement: Supplementary Checklist [file mmc7.docx]

STROBE Statement—checklist of items that should be included in reports of observational studies

|  | Item No. | Recommendation | Page  No. | Relevant text from manuscript |
| --- | --- | --- | --- | --- |
| **Title and abstract** | 1 | (*a*) Indicate the study’s design with a commonly used term in the title or the abstract | 1 | Lines 3-4 |
|  |  | (*b*) Provide in the abstract an informative and balanced summary of what was done and what was found | 2 | Lines 36-62 |
| Introduction | | | |  |
| Background/rationale | 2 | Explain the scientific background and rationale for the investigation being reported | 3-4 | Lines 68-100 |
| Objectives | 3 | State specific objectives, including any prespecified hypotheses | 3-4 | Lines 102-108 |
| Methods | | | |  |
| Study design | 4 | Present key elements of study design early in the paper | 4-6 | Lines 110-186 |
| Setting | 5 | Describe the setting, locations, and relevant dates, including periods of recruitment, exposure, follow-up, and data collection | 4-6 | Lines 110-186 |
| Participants | 6 | (*a*) *Cohort study*—Give the eligibility criteria, and the sources and methods of selection of participants. Describe methods of follow-up  *Case-control study*—Give the eligibility criteria, and the sources and methods of case ascertainment and control selection. Give the rationale for the choice of cases and controls  *Cross-sectional study*—Give the eligibility criteria, and the sources and methods of selection of participants | 4-6 | Lines 110-186 |
|  |  | (*b*) *Cohort study*—For matched studies, give matching criteria and number of exposed and unexposed  *Case-control study*—For matched studies, give matching criteria and the number of controls per case | 4-6 | Lines 110-186 |
| Variables | 7 | Clearly define all outcomes, exposures, predictors, potential confounders, and effect modifiers. Give diagnostic criteria, if applicable | 4-6 | Lines 110-193 |
| Data sources/ measurement | 8* | For each variable of interest, give sources of data and details of methods of assessment (measurement). Describe comparability of assessment methods if there is more than one group | 4-6 | Lines 110-193 |
| Bias | 9 | Describe any efforts to address potential sources of bias | 4s-6 | Lines 110-193 |
| Study size | 10 | Explain how the study size was arrived at | 4-6 | Lines 110-193 |

Continued on next page

| Quantitative variables | 11 | Explain how quantitative variables were handled in the analyses. If applicable, describe which groupings were chosen and why | 4-6 | Lines 110-193 |
| --- | --- | --- | --- | --- |
| Statistical methods | 12 | (*a*) Describe all statistical methods, including those used to control for confounding | 4-7 | Lines 110-206 |
|  |  | (*b*) Describe any methods used to examine subgroups and interactions | 4-6 | Lines 110-193 |
|  |  | (*c*) Explain how missing data were addressed | 4-6 | Lines 110-193 |
|  |  | (*d*) *Cohort study*—If applicable, explain how loss to follow-up was addressed  *Case-control study*—If applicable, explain how matching of cases and controls was addressed  *Cross-sectional study*—If applicable, describe analytical methods taking account of sampling strategy | 4-7 | Lines 110-206 |
|  |  | (*e*) Describe any sensitivity analyses | 4-7 | Lines 110-206 |
| Results | | | | |
| Participants | 13* | (a) Report numbers of individuals at each stage of study—eg numbers potentially eligible, examined for eligibility, confirmed eligible, included in the study, completing follow-up, and analysed | 4-6 | Lines 110-186 |
|  |  | (b) Give reasons for non-participation at each stage | 4-6 | Lines 110-186 |
|  |  | (c) Consider use of a flow diagram | 4-6 | Lines 110-186 |
| Descriptive data | 14* | (a) Give characteristics of study participants (eg demographic, clinical, social) and information on exposures and potential confounders | 7 | Lines 208-217 |
|  |  | (b) Indicate number of participants with missing data for each variable of interest | 4-6 | Lines 110-186 |
|  |  | (c) *Cohort study*—Summarise follow-up time (eg, average and total amount) | NA | NA |
| Outcome data | 15* | *Cohort study*—Report numbers of outcome events or summary measures over time | NA | NA |
|  |  | *Case-control study—*Report numbers in each exposure category, or summary measures of exposure | NA | NA |
|  |  | *Cross-sectional study—*Report numbers of outcome events or summary measures | 4-6  7 | Lines 110-186  Lines 208-217 |
| Main results | 16 | (*a*) Give unadjusted estimates and, if applicable, confounder-adjusted estimates and their precision (eg, 95% confidence interval). Make clear which confounders were adjusted for and why they were included | 7-9 | Lines 208-274 |
|  |  | (*b*) Report category boundaries when continuous variables were categorized | 7-9 | Lines 208-274 |
|  |  | (*c*) If relevant, consider translating estimates of relative risk into absolute risk for a meaningful time period | NA | NA |

Continued on next page

| Other analyses | 17 | Report other analyses done—eg analyses of subgroups and interactions, and sensitivity analyses | 7-9 | Lines 208-274 |
| --- | --- | --- | --- | --- |
| Discussion | | | | |
| Key results | 18 | Summarise key results with reference to study objectives | 9 | Lines 276-284 |
| Limitations | 19 | Discuss limitations of the study, taking into account sources of potential bias or imprecision. Discuss both direction and magnitude of any potential bias | 11 | Lines 336-346 |
| Interpretation | 20 | Give a cautious overall interpretation of results considering objectives, limitations, multiplicity of analyses, results from similar studies, and other relevant evidence | 11 | Lines 336-346 |
| Generalisability | 21 | Discuss the generalisability (external validity) of the study results | 9-11 | Lines 276-355 |
| Other information | |  | | |
| Funding | 22 | Give the source of funding and the role of the funders for the present study and, if applicable, for the original study on which the present article is based | 12 | Lines 377-394 |

*Give information separately for cases and controls in case-control studies and, if applicable, for exposed and unexposed groups in cohort and cross-sectional studies.

**Note:** An Explanation and Elaboration article discusses each checklist item and gives methodological background and published examples of transparent reporting. The STROBE checklist is best used in conjunction with this article (freely available on the Web sites of PLoS Medicine at http://www.plosmedicine.org/, Annals of Internal Medicine at http://www.annals.org/, and Epidemiology at http://www.epidem.com/). Information on the STROBE Initiative is available at www.strobe-statement.org.
